# An Efficient Deep Learning Framework for Revealing the Evolution of Characterization Methods in Nanoscience

**DOI:** 10.1007/s40820-025-01807-z

**Published:** 2025-06-13

**Authors:** Hui-Cong Duan, Long-Xing Lin, Ji-Chun Wang, Tong-Ruo Diao, Sheng-Jie Qiu, Bi-Jun Geng, Jia Shi, Shu Hu, Yang Yang

**Affiliations:** Institute of Artificial Intelligence, Pen-Tung Sah Institute of Micro-Nano Science and Technology, State Key Laboratory of Physical Chemistry of Solid Surfaces, Xiamen University, Xiamen, 361005 People’s Republic of China

**Keywords:** Nanostructure, Deep learning, Data-driven, Raman, Nanoscience

## Abstract

Text mining has emerged as a powerful strategy for extracting domain knowledge structure from large amounts of text data. To date, most text mining methods are restricted to specific literature information, resulting in incomplete knowledge graphs. Here, we report a method that combines citation analysis with topic modeling to describe the hidden development patterns in the history of science. Leveraging this method, we construct a knowledge graph in the field of Raman spectroscopy. The traditional Latent DirichletAllocation model is chosen as the baseline model for comparison to validate the performance of our model. Our method improves the topic coherence with a minimum growth rate of 100% compared to the traditional text mining method. It outperforms the traditional text mining method on the diversity, and its growth rate ranges from 0 to 126%. The results show the effectiveness of rule-based tokenizer we designed in solving the word tokenizer problem caused by entity naming rules in the field of chemistry. It is versatile in revealing the distribution of topics, establishing the similarity and inheritance relationships, and identifying the important moments in the history of Raman spectroscopy. Our work provides a comprehensive tool for the science of science research and promises to offer new insights into the historical survey and development forecast of a research field.

**Supplementary Information:**

The online version contains supplementary material available at 10.1007/s40820-025-01807-z.

## Highlights

- A framework combining the citation analysis with topic modeling is designed to construct the knowledge graph of a research field.
- An extensible tokenizer is designed to improve the universality of the framework, and the performance of topic recognition is superior to that of the traditional method.
- The detailed evolutionary paths of Raman spectroscopy technology are demonstrated, and the significant publications in the Raman spectroscopy are identified.

**Supplementary Information:**

The online version contains supplementary material available at 10.1007/s40820-025-01807-z.

## Graphical abstract

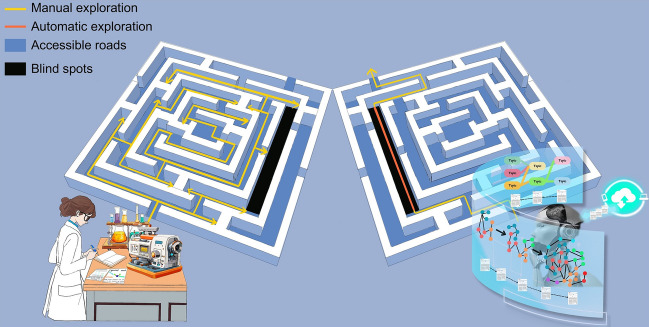

## Introduction

Data-driven methods have attracted much interest in literature survey and fundamental research. They help researchers forecast the hotspots in the near future, and administrators facilitate the formulation of funding policies [1]. As a distributed repository of scientific knowledge, scientific literature represents the fundamental data unit for studying the structure and evolution of science [2]. Traditionally, researchers summarized the patterns and trends of scientific development by reading a large amount of literature one by one. Unfortunately, this paradigm is confronted with unprecedented challenges in the field of nanoscience and nanotechnology. As a field that has attracted much interests from scientists, it usually contains millions of scientific literature, leaving a big challenge to extract research trends and potential research hotspots in nanoscience and nanotechnology manually. To address this issue, in recent years researchers started to utilize quantitative research methods to analyze the evolution of scientific structure and research hotspots, such as literature metrology [3] and science mapping analysis [4]. Nevertheless, most methods rely on particular data formats and literature indicators, resulting in failures to comprehend substantive content and academic ideas.

Text mining methods provide an opportunity for automatically reading literature and extracting the viewpoints therein and are beneficial to reducing time costs and avoiding human errors [5]. The topic models, as generally divided into structural [6], dynamic [7], and neural [8] topic models, had been proven efficient in deducing potential topic distributions and obtaining a birds-eye view of topic evolution [9–11]. The reference section was widely recognized as a significant component of a piece of published literature because it is a complex combination of considerations and it informs the substantial knowledge transfer of important arguments, experimental methods, and discoveries [12]. To date, most of the works were concentrated on the textual information of the literature, paying less attention to considering the inter-reference information. The lack of reference information prevents researchers from delving into the potential connections among literature and results in an incomplete knowledge graph. However, there is a limited method that can incorporate citation information into the topic information in the state-of-art text mining.

Recently, the Bidirectional Encoder Representations from Transformers Topic (BERTopic) model was proposed to generate coherent topic representations [13]. It was a scalable framework that allowed researchers to integrate external information and to construct a complete domain knowledge graph. Herein, we developed a novel method that integrated the BERTopic model and citation analysis to demonstrate the entire evolution of domain knowledge. To get a corpus, the web crawling technique was applied to gather literature from the Web of Science database and the BERTopic model was utilized to extract topics. The traditional approaches like the Latent Dirichlet Allocation (LDA) method were selected as baseline models to demonstrate the performance of our model. A citation network was built using citation information and the community detection algorithm was utilized to determine the correlation between evolution of topic and community structure. Finally, a comprehensive knowledge graph was constructed. As a proof of principle, we employed the field of Raman spectroscopy, a typical characterization method in nanoscience and nanotechnology, to verify the feasibility of our method and demonstrated that our method can identify the important progress of a scientific field hidden in a huge number of literature.

## Materials and Methods

### Model Architecture

**Fig. 1:**
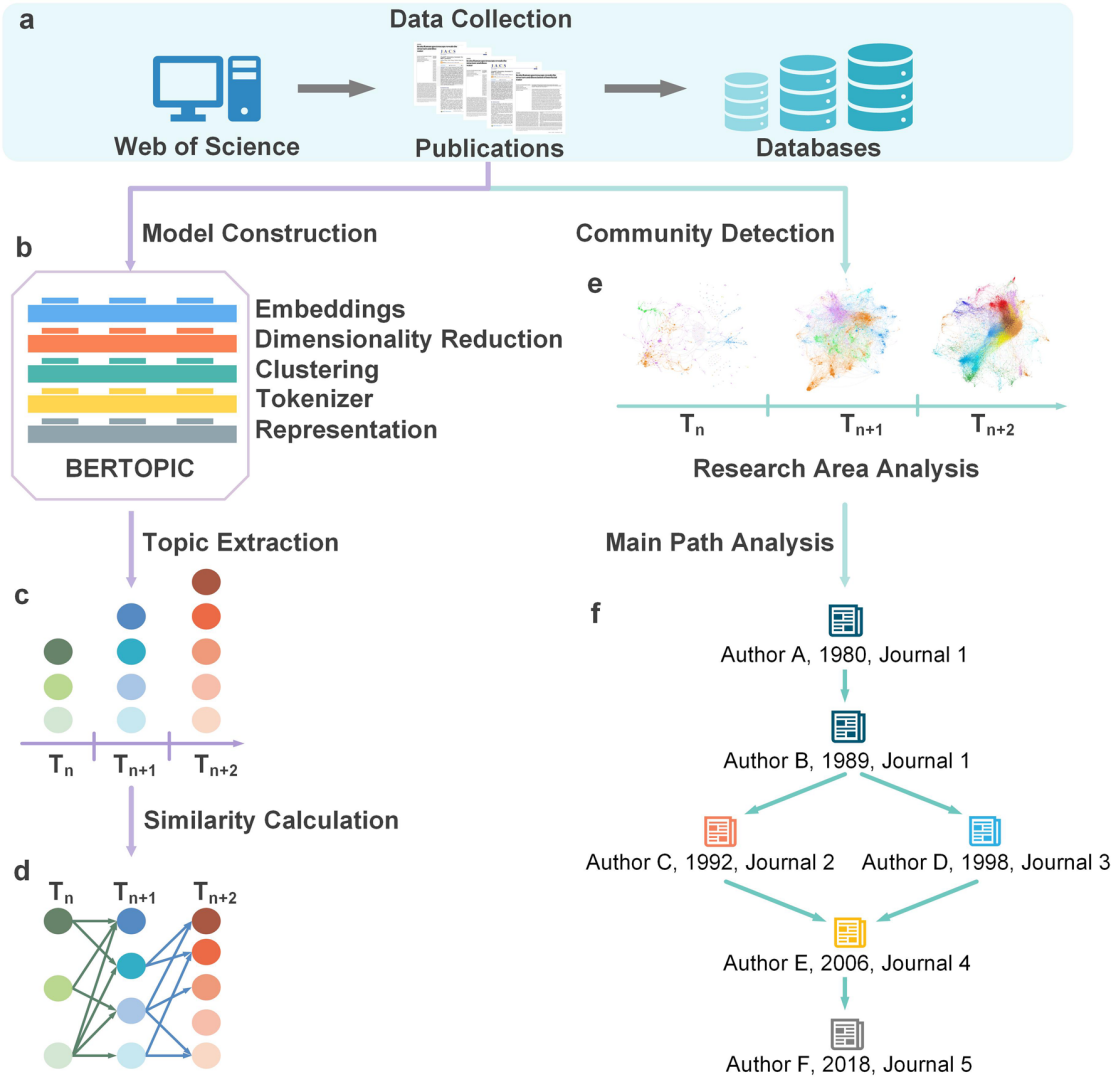
Illustration of model architecture with essential steps and outcomes. **a** Research literature is collected from the Web of Science database as the input of the model. **b** Architecture of our topic model consists of five independent modules. **c** To obtain the distribution of topics in each stage, the topic model is used to extract topics from textual information. **d** Cosine similarity algorithm is applied to get the topic evolution results displayed in a Sankey diagram. **e** Citation network is constructed from the collected research literature and hidden communities are detected by the community detection algorithm. **f** Milestone literature is found through the main path analysis. **a** and **c** represent the input and output of BERTopic model, respectively

The workflow of our method consists of three independent steps: data collection (Fig. 1a), topic model construction (Fig. 1b-d), and citation analysis (Fig. 1e, f). Firstly, the search expression was determined by looking up literature and seeking advice from experts in the field. A large amount of literature was retrieved and collected from the Web of Science through the search expression. Their textual and citation information was stored in our database as a corpus (Fig. 1a).

Secondly, the BERTopic model was constructed to extract topic information from the corpus. As shown in Fig. 1b, the BERTopic model was composed of five modules: embeddings, dimensionality reduction, clustering, tokenizer, and representation. The all-MiniLM-L6-v2 model was chosen to convert a piece of published literature into a 384-dimensional vector in the embeddings module, which could capture the word order and semantic information of input text. The 384-dimensional vector was converted into a five-dimensional vector using the Uniform Manifold Approximation and Projection (UMAP) algorithm in the dimensionality reduction module, which could retain the global data structure as much as possible (Section S1.1). Considering the successful cases of clustering algorithms applied in processing of mass data [14, 15], we integrated a clustering algorithm into BERTopic to improve the computational efficiency (Section S1.2). The reduced vectors were used as the input of the Hierarchical Density-Based Spatial Clustering of Applications with Noise (HDBSCAN) algorithm to discover topic clusters in the clustering module (Table S5). All literature in a topic cluster was merged into a long document in the tokenizer module. The document was then divided into phrase sequences by a tokenizer and the frequency of each phrase was counted. Here, we designed a tokenizer based on the Punkt algorithm [16] and implemented it to make sure that the topic representations fit with domain naming conventions (Section S1.3). Topic terms were identified from phrase sequences using the c-TF-IDF algorithm in the representation module. After extracting topic information (Fig. 1c), the cosine similarity algorithm was applied to establish the evolutionary relationship (Fig. 1d).

Finally, citation information was utilized to construct a citation network. In Fig. 1e, the community detection algorithm was applied to detect the communities hidden in the citation network, which represented specific research areas and academic viewpoints (Section S1.4). Then, as shown in Fig. 1f, the main path analysis algorithm was used to simplify the citation network as it could identify the critical nodes that bridge different research communities.

### Data Collection

We collected literature on Raman spectroscopy from the Web of Science Core Collection. The expressions employed for searching literature on the field of Raman spectroscopy is TS = (Raman *or Raman spectroscopy) AND DT = (Article or Letter or Early Access or Note) AND PY = (1980–2020). The Playwright was employed to crawl the title, keyword, abstract and citation information, and finally 176,008 pieces of literature were obtained. The literature collected was statistically analyzed, and the results are shown in Figs. S13-S16. The dataset covered 122 research areas, demonstrating good disciplinary completeness. About 99% of the literature received 0–50 citations, with 47% cited fewer than 10 times, aligning with typical scientific citation patterns.

### Word Embedding Representation of Literature

The text is converted into numerical vectors in the embedding module of the BERTopic model. Considering the importance of the semantic information among words, the attention mechanism is utilized to preserve the semantic information. However, it takes up a considerable amount of computational resources when the sequence is somehow long. To address this concern, the scaled dot product of pairs of attention head is introduced into our model and its formula is as follows:

1 $$R_{i,j,a}^{t} = softmax \left( {\frac{{A_{i,a}^{t} (A_{j,a}^{t} )^{T} }}{{\sqrt {d_{t} } }}} \right)$$

2 $$R_{i,j,a}^{s} = softmax \left( {\frac{{A_{i,a}^{s} (A_{j,a}^{s} )^{T} }}{{\sqrt {d_{s} } }}} \right)$$

3 $$L_{i,j} = \frac{1}{{A_{r} \left| x \right|}}\sum\limits_{a = 1}^{{A_{r} }} {\sum\limits_{k = 1}^{|x|} {D_{KL} \left( {R_{i,j,a,k}^{t} \left\| {R_{i,j,a,k}^{s} } \right.} \right)} }$$

4 $$L = \sum\limits_{i = 1}^{3} {\sum\limits_{j = 1}^{3} {\alpha_{i,j} L_{i,j} } }$$

where $A_{1,a} ,A_{2,a} ,A_{3,a}$ are the query, key and value of a multiple relation head, respectively, $d_{t}$ is the relation head size of the teacher, $d_{s}$ is the relation head size of the student, $R_{i,j,a}^{t}$ is the self-attention relation between the relation head of teachers, $R_{i,j,a}^{s}$ is the self-attention relation between the relation head of student, $A_{r}$ is the number of relation heads, $\left| x \right|$ is the length of the input sequence and $D_{KL}$ is the Kullback–Leibler Divergence, $L_{i,j}$ is the loss between self-attention relations of the teacher and student, $L$ is the total loss and $\alpha_{i,j}$ is the weight assigned to each self-attention relation loss.

## Results and Discussion

### Division of Development Stages Based on the Lifecycle Theory

The lifecycle theory states that the development of one thing, including the scientific field herein, requires going through stages of emerging, growth, maturity, and decline [17]. In the bibliometrics field, researchers generally employed the growth pattern of published literature to represent the life cycle of the scientific fields. The number of published literature per year in the field of Raman spectroscopy and its first-order derivative curve were displayed in the form of a bar chart and line chart, respectively (Fig. S12). It is noticed that the number of literature was less than 500 per year during the period from 1980 to 1989 and the first-order derivatives of the number of literature were all greater than 0 during the period from 2001 to 2020. The whole period was divided into three stages according to the growth pattern of literature in the field of Raman spectroscopy: T_n_ stage (emerging stage from 1980 to 1989), T_n+1_ stage (growth stage from 1990 to 2000) and T_n+2_ stage (maturity stage from 2001 to 2020).

### Validation of the Topic Extraction Capability of our Model

The LDA model had made significant progress in topic extraction [18]. It was chosen as the baseline model to demonstrate the performance of our method. The Coherence and Diversity metrics were used to measure the quality of the extracted topics. Normalized Pointwise Mutual Information (NPMI) is a coherence indicator that measures the degree of semantic consistency among words, which has been proven to be close to human judgment [19]. Its value ranges from -1 to 1, and the interpretability of the topics is higher when the value is closer to 1. Diversity is applied to estimate the proportion of unique words in a topic, ranging from 0 to 1. The difference among topics is greater when the value is closer to 1. We calculated the NPMI and Diversity in different database sizes and topic numbers to verify the performance and stability of our model. The result is shown in Table 1.

**Table 1:** Performance of LDA and BERTopic models as increased number of topics

Metrics	Database size	Model	TN^[a]^ = 10	TN = 50	TN = 100
NPMI^[b]^	3,126	LDA^[c]^	− 0.03	− 0.08	− 0.08
		BERTopic^[d]^	− 0.12	0.00	− 0.08
		BERTopic_tokenizer	0.08	0.05	0.03
	22,321	LDA	0.03	− 0.08	− 0.13
		BERTopic	0.12	0.10	0.10
		BERTopic_tokenizer	0.10	0.15	0.16
	150,561	LDA	0.06	0.00	− 0.10
		BERTopic	0.14	0.14	0.16
		BERTopic_tokenizer	0.12	0.16	0.19
Diversity	3,126	LDA	0.43	0.38	0.39
		BERTopic	0.95	0.83	0.87
		BERTopic_tokenizer	0.95	0.83	0.88
	22,321	LDA	0.65	0.73	0.73
		BERTopic	0.97	0.83	0.82
		BERTopic_tokenizer	0.99	0.84	0.82
	150,561	LDA	0.79	0.86	0.85
		BERTopic	1.00	0.90	0.84
		BERTopic_tokenizer	0.99	0.90	0.85

^[a]^ TN, ^[b]^ NPMI, ^[c]^ LDA, and ^[d]^ BERTopic are the abbreviations for the number of topics, the Normalized Pointwise Mutual Information, the latent Dirichlet allocation, and the Bidirectional Encoder Representations from Transformers Topic, respectively

When the number of topics (TN) is 10 and the database size is 3,126 and 150,561, our model has the NPMI value of 0.08 and 0.12, which is 367% and 100% higher than that of the LDA model, respectively. When the TN is 100 and the database size is 3,126 and 150,561, the diversity value of our model is 0.88 and 0.85, which is identical to and 126% higher than that of the LDA model, respectively. In totality, our model improves the topic coherence and diversity with a growth rate of 100% to 367% and 0 to 126%, respectively. These indicate our model can extract higher-quality topics than that of the LDA model irrespective of the database size.

To verify the capability of the tokenizer we designed, we analyze the NPMI value in different topic numbers and database sizes. When the database size is 22,321 and the number of topics is 10, 50, and 100, the NPMI values of the BERTopic model are 0.12, 0.10, and 0.10, while the NPMI values of the BERTopic model with our developed tokenizer are 0.10, 0.15, and 0.16, respectively. These results indicate that our tokenizer is effective in improving the interpretability of the topics and is capable of handling domain-specific naming conventions.

### Evolution of Topics Between Adjacent Stages

**Fig. 2:**
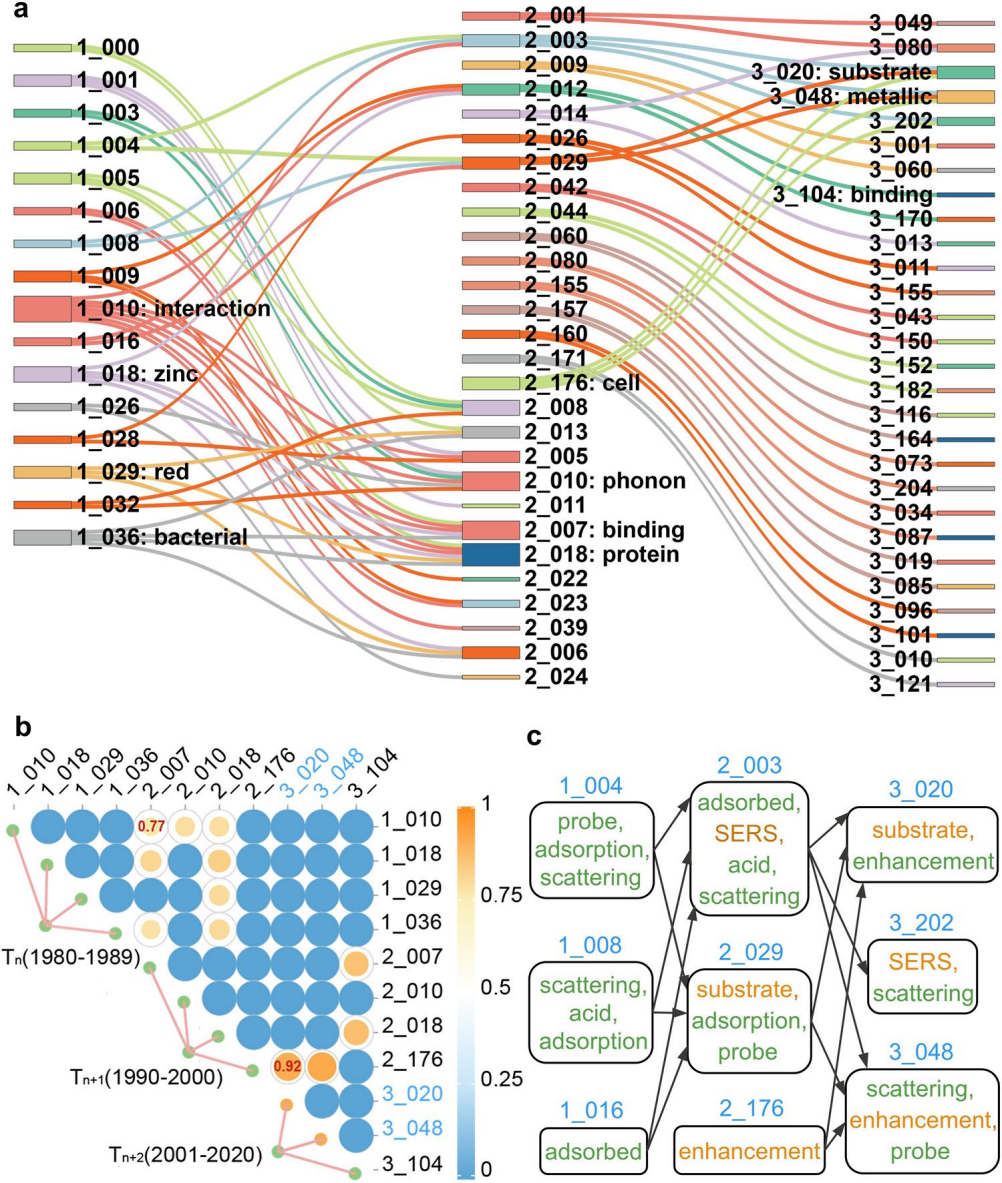
Distribution of topics in different stages of Raman spectroscopy and the evolutionary relationships among topics. **a** Evolutionary relationship among topics with at least two child topics is shown in the form of a Sankey diagram. The label of a topic consists of the stage number and the topic number, separated by an underline. The stage numbers 1, 2, and 3 before underline represent the T_n_, T_n+1_, and T_n+2_ stage, respectively. The topic number indicates the serial number of the topic in the stage. The core topics of each stage are additionally labeled with the most essential topic term. **b** Similarity relationship among core topics is displayed in the form of the correlation heatmap. The minimum and maximum similarity value are 0.77 and 0.92, respectively. **c** Complete evolution paths of topics 3_020 and 3_048 labeled in blue within **b**, and the topic terms corresponding to each topic. The blue words are the label of the node, green words represent the topic terms of the T_n_ stage, and orange words represent the topic terms of the T_n+1_ stage

In science, a research topic is the central issue that researchers pay close attention to and explore in depth. It is a dynamic concept that evolves over time, which is known as topic evolution [20]. When there is an evolutionary relationship between topics in two different stages, they demonstrate significant similarity at the semantic level. To trace and clearly depict the evolution of research topics in the field of Raman spectroscopy over time, we devised a method to calculate the similarity of topics in adjacent stages. The results are presented in Fig. 2.

To demonstrate the core topics in different stages of Raman spectroscopy, we utilized the Sankey diagram to illustrate the distribution of topics in each stage and their evolutionary relationships. In this diagram, if topic a in the previous stage evolves into topics b, c, and d, then a is regarded as the parent topic, while b, c, and d are regarded as child topic accordingly. The occurrence frequency of a topic is the key indicator of the importance of that topic. Based on the figure that completely demonstrated the evolutionary relationship (Fig. S7), we defined core topics as those that have three or more child topics or parent topics and obtained the core research areas of Raman spectroscopy in three different stages (Fig. 2a). It is shown in Fig. 2a that the core topics of the T_n_ stage are topics 1_010, 1_018, 1_029, and 1_036. By analyzing their topic terms (Table S7), we found that topics 1_010, 1_029, and 1_036 were all focusing on the field of biology. Topics 1_029 and 1_036 were closely related to bacteria, which demonstrated the central position of bacterial research in the field of Raman spectroscopy in the T_n_ stage. The core topics of the T_n+1_ stage were topics 2_007, 2_010, 2_018, and 2_176. By analyzing their topic terms, we found that proteins appeared in both topics 2_007 and 2_018, indicating that proteins were the core object of study in this stage. The core topics of the T_n+2_ stage were topics 3_020, 3_048, and 3_104, in which topics 3_020 and 3_048 were related to nanostructured arrays, revealing that the core of the stage was the study of enhancing Raman scattering signals by designing nanostructured arrays, whereas topic 3_104 continued to focus on proteins. Combined with the previous topics, we found that the research on proteins has been carried out throughout the development of Raman spectroscopy.

To elucidate the correlation and evolutionary trends between the core topics in different research stages, we presented the correlation heatmap in Fig. 2b, which visualized the similarity that existed among the core topics. It is noteworthy that the core topics of stage T_n+1_ and stage T_n+2_ exhibited a higher similarity in comparison to those of stage T_n_ and stage T_n+1_. This trend held for all topics in Fig. 2b (Figs. S17 and S18). This phenomenon indicated that the knowledge gained in previous stages was effectively integrated and absorbed, resulting in a concentration of research focus and a relatively smooth development of research direction. The highest similarity between topics 2_176 and 3_020 with a value of 0.92 indicated that the knowledge had been directly transferred and the research direction had been further developed, which was additional supported by their topic terms. Topic 2_176 was concerned with the general principles of surface-enhanced Raman spectroscopy (SERS), and topic 3_020 explored the design of specific nanostructure (Table S7). The lowest similarity was observed between topics 1_010 and 2_007, with a value of 0.77. The comparison of the topic terms revealed that although they were both concerned with research of Raman spectroscopy and protein, the research directions were quite different. Topics 1_010 and 2_007 were more focused on the basic physicochemical study of proteins and development of optical enhancement techniques and specific application, respectively.

To demonstrate the connection between evolutionary relationships and topic terms, we took the complete evolutionary path of topics 3_020 and 3_048 in the T_n+2_ stage as an example. As shown in Fig. 2c, topics 2_003 and 2_029 in the T_n+1_ stage were formed by combining topics 1_004, 1_008, and 1_016 in the T_n_ stage. They were both related to molecular adsorption, and their common topic terms are “surface-enhanced Raman” and “SERS” (Table S7). The difference was that the topics in the T_n_ stage focused on the surface-enhanced Raman effect and characteristics of traditional metal materials such as silver and gold, whereas the topics in the T_n+1_ stage paid attention to the surface-enhanced Raman scattering properties of new materials such as nanoparticles and photocatalytic materials, involving more advanced spectroscopic techniques such as near-infrared spectroscopy and resonance Raman scattering. The topics in the T_n+2_ stage were formed by the fusion of topics 2_003, 2_029, and 2_176 in the T_n+1_ stage. Topic 3_020 inherited topic terms from its parent topics, such as “substrates” and “enhancement”. Based on the research foundation of the parent topics, topics 3_020 and 3_048 further investigated the Raman enhancement effect on large-scale nanostructured array substrates. All of these indicate the emergence of evolutionary relationships is accompanied by the inheritance of topic terms.

### Evolution of the Academic Communities Extracted by the Louvain Algorithm

To build a complete knowledge graph, we constructed a citation network in the field of Raman spectroscopy. In this citation network, literature is naturally clustered because all of them belong to a research topic, forming an academic community [21]. The community density is an indicator that assesses the proximity of node connections within an academic community [22]. It provides insight into the efficiency of knowledge transfer within the community [23]. To investigate the relationship between the topic evolution and community density in the field of Raman spectroscopy, we employed the Louvain algorithm to identify hidden academic communities in the citation network and investigated the change in the density of each community in different stages.

**Fig. 3:**
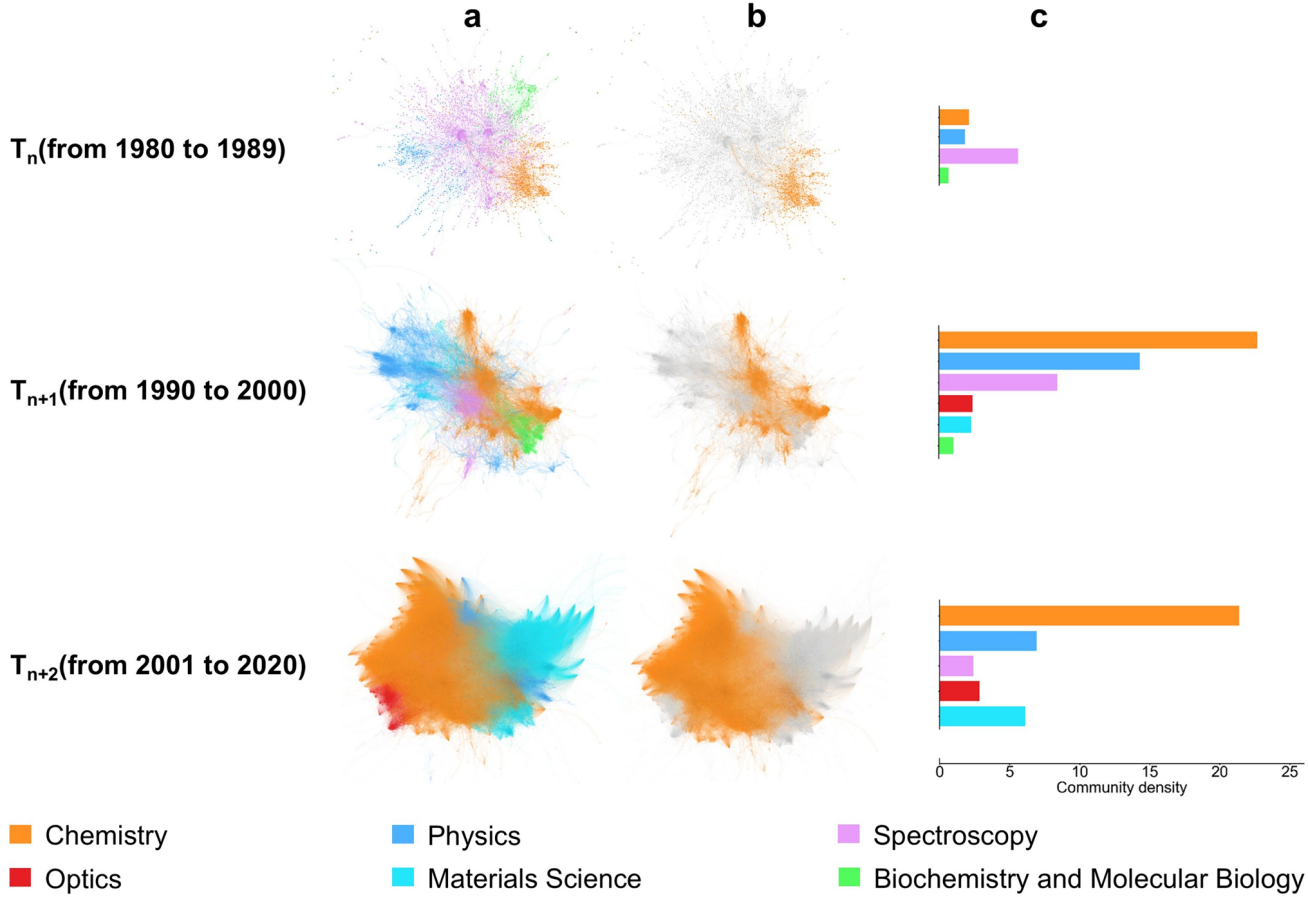
Distribution and density evolution of academic communities in different stages in the field of Raman spectroscopy. **a** Academic communities in the field of Raman spectroscopy were identified by the Louvain algorithm and marked with different colors. The distribution of academic communities is shown from 1980 to 1989 (Upper), 1990 to 2000 (Middle), and 2001 to 2020 (Lower), respectively. **b** Distribution of Chemistry communities in the field of Raman spectroscopy is shown from 1980 to 1989 (Upper), from 1990 to 2000 (Middle), and from 2001 to 2020 (Lower), respectively. The percentage of nodes for Chemistry community in the citation network at each stage are 16.40%, 36.56%, 57.61%, respectively. **c** Distribution of community density in different stages is shown in the form of histogram. The color of the histogram is consistent with the corresponding academic community color in the citation network

We reported the distribution of academic communities in different stages in the field of Raman spectroscopy. In the T_n_ stage, four communities were identified within the citation network, namely Spectroscopy, Chemistry, Biochemistry and Molecular Biology, and Physics (Fig. 3a). The Spectroscopy community exhibited the highest density value of 5.69, while the Biochemistry and Molecular Biology community demonstrated the lowest density value of 0.67 (Fig. 3c). In the T_n+1_ stage, several new academic communities emerged, including those related to Materials Science and Optics (Fig. 3a). The Optics community had the highest density value of 2.29 among the emerged academic communities in T_n+1_ stage (Fig. 3c). In the T_n+2_ stage, the number of nodes increased rapidly, with nearly 99% of nodes in the citation network belonging to the four communities of Chemistry, Materials Science, Physics, and Optics (Fig. 3a). This resulted in the overshadowing of newly emerged academic communities, such as the Mineralogy community, the Toxicology community and the Astronomy and Astrophysics community.

To investigate the intrinsic relationship between the evolution of the topic and community density, we analyzed the evolution of community density in different stages and the Chemistry community was selected as a case study. Figure 3b illustrates the evolution of Chemistry communities from T_n_ stage to T_n+2_ stage. The node proportion of Chemistry community demonstrated a gradual increase in the citation network, reaching a maximum value of 57.61% in the T_n+2_ stage. This trend indicated that the Chemistry community had become a central focus in Raman spectroscopy and increasingly demonstrated its significance in applications. The increase in the proportion of nodes within the citation network was accompanied by the dynamic shift of the density value of Chemistry community. The lowest density value was 2.16 in the T_n_ stage, while the highest density value was 22.70 in the T_n+1_ stage (Fig. 3c). This evolution was indicative of the growing concentration of research related to Raman spectroscopy within the field of chemistry, as well as the increasing dissemination of knowledge during this stage. It is consistent with the trend of topic similarity shown in Fig. 2b. It can also be observed from Fig. 2c. For example, the term “adsorption” is a common topic term for topics 1_004, 1_008, and 1_016 in T_n_ stage, whereas “enhancement” is a common topic term for topics 3_020 and 3_048 in T_n+2_ stage.

### Reasons for the Evolution of Topic and Academic Community

The previous analyses have provided some insight into the evolution of topic and academic community in the field of Raman spectroscopy, leading to a preliminary macro-picture of the history of Raman spectroscopy. However, the key factors driving these evolutions remain to be explored. Therefore, we combined these existing understandings with the main path analysis method to reveal the historical trajectory of Raman spectroscopy more clearly. It is possible to make evidence-based predictions about the future development of the field based on this.

**Fig. 4:**
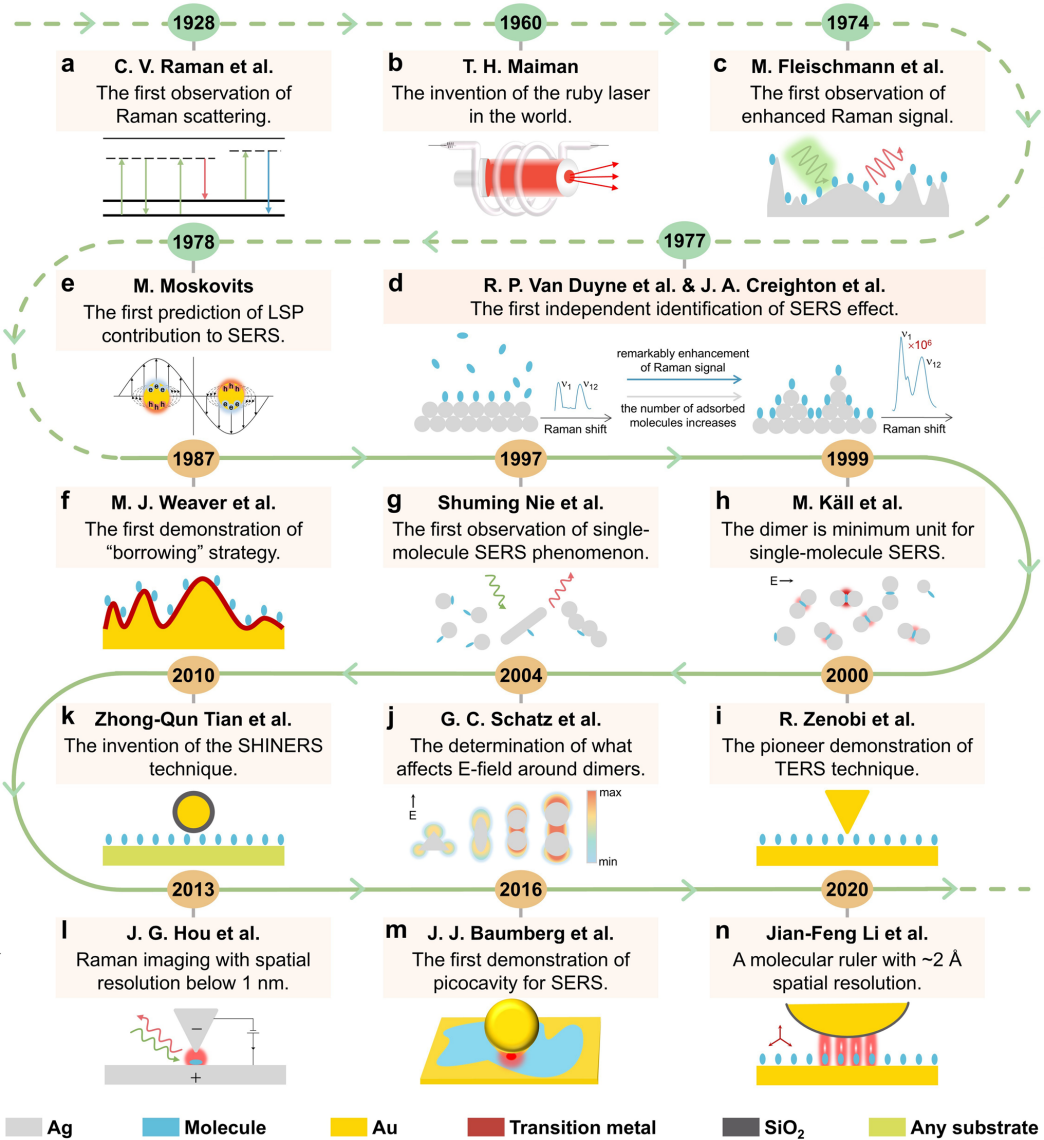
Milestone literature in the field of Raman spectroscopy identified by the citation network and main path analysis. **a-n** Milestone literature are represented in the form of nodes with the publication year, the last corresponding author and highlights of the literature. Nodes are ordered by the publication year. The nodes on the dotted line are also extremely significant in the history of Raman spectroscopy, but are not included in the web of science because their publication year are before 1980. The abbreviations in this figure: surface-enhanced Raman spectroscopy for SERS, localized surface plasmon for LSP, shell-isolated nanoparticle-enhanced Raman spectroscopy for SHINERS, tip-enhanced Raman spectroscopy for TERS, electric field for E-field

To demonstrate the reason for the evolution, we used the main path analysis method and found the inevitable paths for the knowledge flow in the field of Raman spectroscopy (Sections S1.5 and S1.6). The results were shown in the global main path graph (Fig. S10) and node centrality table (Tables S3 and S4), where nodes are related to either technological or application breakthroughs in the field of Raman spectroscopy. With these nodes, the history of Raman spectroscopy is demonstrated from a technical perspective. The Web of Science database only collected research literature published after 1980, but the Raman scattering is discovered in 1928. To completely demonstrate the technological trajectory of Raman spectroscopy, five nodes were added to Fig. 4, which were milestone publications published before 1980. These nodes were sorted by publication year and the result is shown in Fig. 4.

Taking Fig. 4 and the results of topic evolution at hand, we were able to clearly trace the historical development process of Raman spectroscopy. In 1928, C.V. Raman et al. [24] reported the first experimental observation of Raman scattering (Fig. 4a), a phenomenon that laid the foundation for the development of Raman spectroscopy technique. Unfortunately, the Raman signal was weak because of using mercury arc lamps with poor light intensity as the excitation light source between the year of 1928 and 1960. The invention of the ruby laser, reported by T.H. Maiman [25] in 1960, greatly advanced the application of Raman spectroscopy (Fig. 4b). It provided an excitation source with excellent coherence, which significantly enhanced the intensity of the Raman signal. The first observation of enhanced Raman scattering from a roughened metal surface was reported by M. Fleischmann et al. [26] in 1974 (Fig. 4c), laying the foundation for a new technique known as SERS. R P. Van Duyne et al. [27] and J.A. Creighton et al. [28] reported the first independent identification of SERS effect in 1977 (Fig. 4d). In 1978, M. Moskovits [29] firstly introduced localized surface plasmon to explain SERS effect (Fig. 4e), which was later called electromagnetic mechanism (EM). Fourteen years later, A. Otto et al. [30] revealed that the electron-mediated resonance Raman effects in metals arose from enhanced electron–photon coupling at the rough metal surface and transient charge transfer to the orbitals of the adsorbate, a process now recognized as the chemical mechanism (CM). This literature was omitted by our method because it was a piece of review literature Review literature is excluded because they mainly synthesize existing knowledge rather than contributing originally experimental findings. It is now widely accepted that the enhancement mechanism of SERS is the result of a combination effect of EM and CM.

According to the content of the nodes identified by our algorithm in Fig. 4, they mainly focused on solving three major problems in the development of Raman spectroscopy: substrate and material universality, sensitivity enhancement, and spatial resolution improvement. Before the 1980s, the detection of Raman signals was predominantly confined to a limited type of materials, such as gold, silver, and copper. Consequently, many scholars worldwide initiated investigations into the feasibility of Raman spectroscopy experiments on metals other than those previously mentioned. Our algorithm identified two pieces of seminal literature among the numerous research literature that emerged. The first literature, with a weighted centrality of 1.41, was published by M.J. Weaver et al. in 1987 [31]. They worked along the borrowing strategy and reported the first demonstration of the “borrowing” strategy (Fig. 4f). Meanwhile, a series of works focused on surface electrochemical roughening had been carried out by the group of Zhong-Qun Tian. They were able to successfully obtain SERS signals from a few metals for which could not be obtained using conventional Raman spectroscopy, including Pt [32, 33], Fe [34], Ni [35], and others [36, 37]. Combined with topic 1_026 (study of copper-based thin film materials) and topic 2_024 (study of nickel materials) in Fig. 2a, we believed that the results of these researches were parts of the driving forces for the evolution of topic 1_026 in the T_n_ stage to topic 2_024 in the T_n+1_ stage. Although these studies had broadened SERS to various transition metals, there were still many other types of non-metallic materials that were not applicable for excitation of the Raman effect. Our algorithm also identified another key literature with a weighted centrality of 41.80, published by Zhong-Qun Tian et al. in 2010 (Fig. 4k), which reported the invention of the shell-isolated nanoparticle-enhanced Raman scattering (SHINERS) technique [38]. It fundamentally solved the bottleneck of SERS substrate and surface topography versatility and promoted the application of Raman spectroscopy in the fields of materials science, food safety, and environmental pollutant detection [39].

Achieving single-molecule detection is the goal of Raman spectroscopy in sensitivity, and our algorithm identified three pieces of significant literature from numerous research findings. The first literature had the highest weighted centrality of 400.41 in the citation network of Raman spectroscopy, which was published in 1997 by Shuming Nie et al. [40] and reported the first observation of the single-molecule SERS phenomenon (Fig. 4g), implying that the sensitivity of SERS reached the single-molecule level. This result not only caused a great sensation at that time, but also remained one of the important cornerstones of research in this field. The second innovative literature had a weighted centrality of 36.32 and was published by M. Käll et al. in 1999 [41], which reported that the dimer was minimum unit for single-molecule SERS (Fig. 4h) and explained the main mechanism of single-molecule SERS experimentally. The third seminal literature with a weighted centrality of 6.56 was published by G.C. Schatz et al. in 2004 [42], which reported the determination of what affects E-field around dimers (Fig. 4j), providing the great potential of the dimer of Ag triangular prisms in single-molecule SERS studies. The single-molecule SERS provided an excellent tool for life science and single-molecule study, increasing the density of biochemistry and molecular biology, from 0.67 in the T_n_ stage to 1.03 in the T_n+1_ stage (Table S6).

The improvement of spatial resolution has constituted a significant challenge in the advancement of Raman spectroscopy. Our algorithm identified four pieces of important literature from many relevant research findings, which were presented according to their publication dates. The first literature had a weighted centrality of 11.16 and was published by R. Zenobi et al. in 2000 [43], which reported the pioneer demonstration of tip-enhanced Raman spectroscopy (TERS) technique (Fig. 4i). TERS is one of the two most important variants of SERS, which improved the lateral resolution of Raman spectroscopy up to 55 nm. The second literature had a weighted centrality of 7.05 and was published by J. G. Hou et al. in 2013 [44], which reported Raman imaging with spatial resolution below 1 nm (Fig. 4l) at cryogenic environment, thereby providing a new method for the study of nonlinear optical processes on a single-molecule scale. The third literature had a weighted centrality of 0.96 and was published by J.J. Baumberg et al. in 2016 [45], which reported the first demonstration of picocavity for SERS (Fig. 4m), paving the way for atomic-scale optical experiments. The last literature had a weighted centrality of 0.91 and was published by Jian-Feng Li et al. in 2020 [46], which reported a molecular scale with ~ 2 Å spatial resolution (Fig. 4n), enriching our understanding of plasma exciton fields. These above studies improved the resolution of Raman spectroscopy and promoted its application in other fields, such as food safety and energy [47, 48].

## Conclusions

In summary, we developed a generic approach based on topic modeling and citation networks to construct a complete domain knowledge graph from numerous literature. We conducted a case study in the field of Raman spectroscopy and collected related literature from Web of Science as the dataset to assess the effectiveness of our method. Performance comparison results showed that our method outperformed the LDA model in terms of topic coherence and topic diversity. Notably, the performance improvement ranged from 100% to 367% and from 0% to 126% in terms of topic coherence and topic diversity, respectively. These results showed that our method was capable of capturing the intricate semantic structure in text. The topic evolution results revealed the topic distribution and evolutionary relationships of Raman spectroscopy, highlighting that Raman spectroscopy research was deepening and expanding, which was externally manifested in the inheritance of topic terms. The results of the citation analysis not only revealed the distribution characteristics of the academic community in the field of Raman spectroscopy, but also demonstrated that the density fluctuation trend of the academic community was highly consistent with the results of the topic evolution. To study the driving forces behind the evolution trends, we introduced the main path analysis algorithm. By analyzing the literature identified by the algorithm, we found that they corresponded to literature that was widely considered to be milestone literature in the field of Raman spectroscopy, which revealed the reasons for evolution in research trends and were important support for the topic evolution. This work provided a convenient and interpretable method for extracting hidden patterns of field development across scientific fields.

## Supplementary Information

Below is the link to the electronic supplementary material.

Supplementary file1 (DOCX 10262 KB)
