# Supplementary material for: An Efficient Deep Learning Framework for Revealing the Evolution of Characterization Methods in Nanoscience
Source: Nanomicro Lett. 2025 Jun 13;17:295. doi: 10.1007/s40820-025-01807-z (PMC12165910; doi:10.1007/s40820-025-01807-z)
Supplement: Supplementary file 1 — Supplementary file1 (DOCX 10262 KB) [file 40820_2025_1807_MOESM1_ESM.docx]

Supporting Information for

**An Efficient Deep Learning Framework for Revealing the Evolution of Characterization Methods in Nanoscience**

Hui-Cong Duan^1^, Long-Xing Lin^1^, Ji-Chun Wang^1^, Tong-Ruo Diao^1^, Sheng-Jie Qiu^1^, Bi-Jun Geng^1^, Jia Shi^1^, Shu Hu^1^, Yang Yang^1^*

^1^ Institute of Artificial Intelligence, Pen-Tung Sah Institute of Micro-Nano Science and Technology, State Key Laboratory of Physical Chemistry of Solid Surfaces, Xiamen University, Xiamen 361005, China

* Correspondence author. E-mail: [yangyang@xmu.edu.cn](mailto:yangyang@xmu.edu.cn) (Yang Yang)

# S1 Supplementary Methods for BERTopic and the Main Path Analysis Algorithm

## S1.1 UMAP algorithm

In the dimensionality reduction module of the BERTopic model, the UMAP algorithm was used to solve the problem of high clustering costs caused by the high dimensionality of word vectors. The dimensionality reduction process in UMAP is divided into graph construction and layout. In the graph construction step, a weighted and undirected K-neighborhood graph is constructed to learn the manifold structure of the high-dimensional space. In the graph layout step, the K-neighborhood graph is mapped to a low-dimensional space. The cross-entropy is used as the objective function and minimized via the stochastic gradient descent method to get the best dimensionality reduction result. This made the fuzzy topological structure between the reduced-dimensional and original data as similar as possible.

The detailed calculation process for each stage is described below. In the graph construction step, the input is $X=\{x_{1},x_{2},x_{3,}...,x_{N}\},x_{i}\in R^{M}$. For each $x_{i}$, we defined its nearest neighbor distance $\rho_{i}$ via:

$\rho_{i}=\min\{d(x_{i},x_{j})|1\leq j\leq k\},$ [S1]

where $k$ is the number of nearest points of $x_{i}$, $d(x_{i},x_{j})$is the distance between $x_{i}$ and $x_{j}$. Then, we used $\rho_{i}$ to solve $\sigma_{i}$ from the following equation, which was the normalization factor of distance and defined the local Riemann metric of $x_{i}$ [S1].

$\log_{2}(k)=\sum_{j=1}^{k} e^{(\frac{-d(x_{i},x_{j})-\rho_{i}}{\sigma_{i}})},$ [S2]

To get a weighted graph $\bar{G}$, the $\rho_{i}$ and $\sigma_{i}$ were used to get the weight of an edge:

$w((x_{i},x_{j}))=e^{(\frac{-d(x_{i},x_{j})-\rho_{i}}{\sigma_{i}})},$ [S3]

where $(x_{i},x_{j})$ is a directed edge pointing from $x_{i}$ to $x_{j}$. The weighted graph we got was $\bar{G}=(V,E,W)$, where $V=X$ and $E=\{(x_{i},x_{j})|1\leq i\leq N,1\leq j\leq k\}$. The graph $\bar{G}$ was a directed graph with misaligned edge weights, so it was unable to be transformed into an undirected graph. To address this issue, the symmetric normalized Laplacian matrix of $\bar{G}$ was calculated via:

$G=D^{\frac{1}{2}}(D-A)D^{\frac{1}{2}},$ [S4]

where $A$ is the weighted adjacency matrix of $\bar{G}$, and $D$ is the degree matrix of $\bar{G}$.

In the graph layout step, the initial dimensionality reduction data was:

$\lambda_{L}=[\lambda_{1},\lambda_{2},\lambda_{3},...,\lambda_{d}]=\left[\begin{matrix} y_{1} \\ y_{2} \\ y_{3} \\ ... \\ y_{N} \end{matrix}\right],\lambda_{L}\in R^{N\times d},$ [S5]

$Y=\{y_{1},y_{2},y_{3},...,y_{N}\},y_{i}\in R^{d},$ [S6]

where $\lambda_{i}\in R^{N}$ is an eigenvector of $G$, $d$ is the desired data dimensions, $\text{y}_{i}$ is the initial data of $x_{i}$ in low dimensional space and $Y$ is a set of input points mapped to the low dimensional space. To keep the fuzzy topological structure, the cross-entropy was used as a loss function, which could effectively measure the difference between two distributions [S2].

$C_{UMAP}=\sum_{\begin{matrix} i\neq j \\ i,j\in V \end{matrix}} v_{ij}\log_{2}(\frac{v_{ij}}{w_{ij}})+(1-v_{ij})\log_{2}(\frac{1-v_{ij}}{1-w_{ij}}),$ [S7]

$w_{ij}=\frac{-2a_{2}b_{2}||y_{i}-y_{j}||_{2}^{2(b-1)}}{\text{1+||y}_{i}-y_{j}||_{2}^{2}}w((x_{i},x_{j}))(y_{i}-y_{j}),$ [S8]

where $a_{2}$ and $b_{2}$ are hyper-parameters, and their values can be seen in Table S1, $||y_{i}-y_{j}||_{2}^{2}$ is the square of the Euclidean norm of $y_{i}$ and $y_{j}$, $w_{ij}$ is the weight value of the edge $(x_{i},x_{j})$ in low dimensional space, $v_{ij}$ is the weight value of the edge $(x_{i},x_{j})$ in high dimensional space and $C_{UMAP}$ is the cross-entropy loss function. Then, to get the best dimensionality reduction result, the stochastic gradient descent method was applied to optimize the cross-entropy loss function.

## Table S1 Hyper-parameter corresponding to each algorithm

| Algorithm | Hyper**-**Parameter | Description |
| --- | --- | --- |
| UMAP | $k=15$ | the nearest number of a point |
|  | $d=5$ | desired data dimensions |
|  | $a_{2}=1.929$ | a hyper-parameter for calculating edge weights in low dimensional space |
|  | $b_{2}=0.7915$ | a hyper-parameter for calculating edge weights in low dimensional space |
| HDBSCAN | $min\_size=15$ | the minimum number of samples in a cluster |
| main path analysis | $h=0.85$ | the weight value of indirect reference |
| topic evolution | $\varepsilon=0.80$ | a threshold for determining the topic evolution relationship between the T_n_ and T_n+1_ stage |
|  | $\varepsilon_{1}=0.88$ | a threshold for determining the topic evolution relationship between the T_n+1_ and T_n+2_ stage |
| LDA | alpha=‘symmetric’ | the prior belief on document-topic distribution and ‘symmetric’ means the usage of the fixed symmetric prior of ‘1.0 / num_topics’ |
|  | decay=0.7 | a value between (0.5, 1], serving as a factor to determine the discarded percentage of the prior lambda value |
|  | iterations=50 | the maximum value of iterations through the corpus for each new examined document when inferring the topic distribution of a corpus |

The UMAP algorithm retains the global data structure as much as possible with no limit on embedding dimensions, and can be extended to larger dimensional data sets. Therefore, we applied the UMAP algorithm to reduce the dimension of word embedding. After multiple attempts, we got the best dimensionality reduction effect when $k$ and $d$ was set to 15 and 5, respectively.

## S1.2 Hierarchical Density-Based Spatial Clustering of Applications with Noise

The clustering algorithm implemented in our model is Hierarchical Density-Based Spatial Clustering of Applications with Noise (HDBSCAN). The process begins by calculating the k-th nearest neighbor distance for each data point to construct a mutual reachability distance matrix between all pairs of points, as formally defined in Equations [S9] and [S10]:

$core_{k}(y)=d(y,N^{k}(y))$ [S9]

$d_{mreach-k}(a,b)=max\{core_{k}(a),core_{k}(b),d(a,b)\}$ [S10]

where $N^{k}(y)$ represents the k-th nearest neighbor point to sample y, $core_{k}(y)$ represents the k-th core distance. $d(a,b)$ represents the original pairwise distance between sample points.

Subsequently, a minimum spanning tree (MST) is constructed and hierarchical cluster structures are established based on mutual reachability distances, ultimately capturing latent thematic clusters embedded within the document collection. The formal equations are defined as follows:

$\lambda=\frac{1}{distance}$ [S11]

$s_{cluster}=\sum_{p\in cluster} (\lambda_{p}-\lambda_{birth})$ [S12]

where $\mathbf{s}_{\mathbf{cluster}}$ represents the measurement for the stability of each cluster, $\mathbf{\lambda}_{\mathbf{p}}$ represents the value when separating the current node from the current cluster, $\mathbf{\lambda}_{\mathbf{birth}}$ is the value at the time of cluster $\lambda$ formatting.

## S1.3 Tokenizer

In the tokenizer module of the BERTopic model, we designed a tokenizer to obtain a list of phrases in the document and the frequency of each phrase. The tokenization process converts a text paragraph into a list of phrases or words. The first step is to divide the paragraph into sentences. Generally, a period marks the end of a sentence in text paragraphs. However, text paragraphs in many scientific fields contain abbreviations, which include periods internally or at the end. This reduces the accuracy of sentence segmentation. Here, we designed a tokenizer based on the Punkt algorithm. It sets three criteria that accurately identify abbreviations, and these criteria are independent of context and language category [S3]. With this algorithm, we accurately segmented sentences without labeled training data.

The second step is to convert sentences into tokens corresponding to phrases, words, or punctuation marks. Researchers commonly employ particular characters as delimiters among tokens, such as spaces, hyphens, commas, or parentheses. However, this approach is impractical in chemistry, as the name of the chemical entity often contains these delimiters. Chemical documents also contain special symbols, numbers, and units of measurement, which also affect the performance of the tokenizer. To solve this problem, we designed a rule-based tokenizer with excellent scalability, which accurately segments tokens based on predefined grammar rules.

Table S2 shows some of the rules we established: (1) If the SPLIT markers occur in a token, we should divide the token before and after these characters wherever they appear, unless the entire token is one of these markers. (2) If a token ends with one of the SPLIT_END markers, it must be partitioned before these markers. (3) If there are no numbers before the SPLIT_END_NO_DIGIT markers, we must continue splitting the token. (4) If the token starts with the NO_SPLIT_PREFIX markers, we do not need to split the token before and after the hyphen. (5) If the token starts with the SPLIT_SUFFIX markers, we need to split the token before and after the hyphen. (6) We use a regular expression to determine whether the token combines numbers and units of measure.

When the number of topics is small, the expanded document coverage per topic causes semantic overgeneralization, and cross-domain terms with frequent occurrence are more likely to become topics. Thus domain-specific terms for differentiating distinct topics are marginalized due to their infrequent occurrence.

## Table S2 Example of syntax rules

| Name | Example |
| --- | --- |
| SPLIT | ‘----’, ‘––––’, ‘————’, ‘<--->‘, ‘---’, ‘–––’, ‘———’, ‘<-->’, ‘-->’, ‘...’, ‘--’, ‘––’, ‘——’ |
| SPLIT_END | ‘:’, ‘,’, ‘(TM)’, ‘(R)’, ‘(®)’, ‘(™)’, ‘(■)’, ‘(◼)’, ‘(●)’, ‘(▲)’, ‘(○)’, ‘(◆)’ |
| SPLIT_END_NO_DIGIT | ‘(aq)’, ‘(aq.)’, ‘(s)’, ‘(l)’, ‘(g)’ |
| NO_SPLIT_PREFIX | ‘e’, ‘a’, ‘u’, ‘x’, ‘agro’, ‘ante’, ‘anti’, ‘arch’, ‘be’, ‘bi’, ‘bio’, ‘co’, ‘counter’, ‘cross’, ‘cyber’, ‘de’, ‘eco’, ‘ex’, ‘extra’, ‘inter’, ‘intra’, ‘macro’, ‘mega’, ‘micro’, ‘mid’, ‘mini’, ‘multi’, ‘neo’, ‘non’, ‘over’, ‘pan’, ‘para’, ‘peri’, ‘post’, ‘pre’, ‘pro’, ‘pseudo’, ‘quasi’, ‘re’, ‘semi’, ‘sub’, ‘super’, ‘tri’, ‘ultra’, ‘un’, ‘uni’, ‘vice’, |
| SPLIT_SUFFIX | ‘absorption’, ‘abstinent’, ‘abstraction’, ‘abuse’, ‘accelerated’, ‘accepting’, ‘acclimated’, ‘acclimation’, ‘acid’, ‘activated’, ‘activation’, ‘active’, ‘activity’, ‘addition’, ‘adducted’, ‘adducts’, ‘adequate’, ‘adjusted’, ‘administrated’, ‘adsorption’, ‘affected’, ‘aged’, ‘alcohol’, ‘alcoholic’, ‘algae’, ‘alginate’, ‘alkaline’, ‘alkylated’, ‘alkylation’, ‘alkyne’, ‘analogous’, ‘anesthetized’, ‘appended’, ‘armed’, ‘aromatic’, ‘assay’, ‘assemblages’, ‘assisted’, ‘associated’, ‘atom’, ‘atoms’, ‘attenuated’, ‘attributed’, ‘backbone’ |
| regular expressions | ‘^((\d\d\d)g|((-−) \d+\.\d+|10(-−)\d+)(g|s|m|N|V)((-−) (1-4)) |(\d*(-−) \d+\. \d*)((pnµμm)A|(µμmk)g|(kM)J|m(lL)|(nµμm) M|(nµμmc)m|kN|(mk)V|(mkMG) W|(mnpμµ)s|Hz|(Mm)(Oo)(Ll)(e|ar) s |k Pa|ppm|min)((-−) (1-4)) )$’ |

## S1.4 Louvain algorithm

Community detection refers to dividing nodes into multiple communities within a given network, and a node can belong to multiple communities simultaneously. In the community detection step of our model, the community detection algorithm was utilized to detect the communities hidden in the citation network, which represented specific research areas and academic viewpoints. The Louvain algorithm is a classic community detection algorithm based on modularity [S4]. Modularity is defined explicitly as follows:

$Q=\frac{1}{2m}\sum_{i,j} (A_{i,j}-\frac{k_{i}k_{j}}{2m})\delta(c_{i},c_{j}),$ [S13]

where $Q$ is the modularity, $m$ is the number of edges in this network, $k_{i}$ is the degree of the node $i$, and $A_{i,j}$ is the adjacency matrix. If node $i$ and node $j$ are in the same community, $\delta(c_{i},c_{j})$ is 1. Otherwise, $\delta(c_{i},c_{j})$ is 0.

Firstly, each node in a network label itself as its own community label. Each node merges with its adjacent nodes in sequence, and then calculates whether their maximum modularity gain is greater than 0. If the maximum modularity is greater than 0, the node is placed in the community where the adjacent node with the maximum modularity gain is located. Repeat This step will be repeated until the modularity does not increase while changing the community label of any nodes. Then, nodes with the same community label are merged into a new node, whose edge weight is the sum of the edge weights of all nodes in the original community, forming a new network. The above steps will be repeated in this new network until the algorithm stabilizes.

## S1.5 The main path analysis algorithm

In the main path analysis step of our model, the main path analysis algorithm was used to simplify the citation network and find the knowledge flow path that could not be ignored in the development of the field. The algorithm is divided into two steps: assigning weight and searching. In the assigning weight step, we need to assign a corresponding weight value to each edge of the citation network. Here, we defined the weight value as follows:

$weight(e_{i})=h\frac{in_{e_{i}}}{in_{\max\_edge}}+(1-h)\frac{c_{ns}+c_{ne}}{c_{\max\_node}},$ [S14]

where $e_{i}$ refers to the edge and $in_{e_{i}}$ is the number of indirect references of $e_{i}$, $in_{\max\_edge}$ is the maximum number of indirect references in the network, $c_{ns}$ and $c_{ne}$ are the number of direct references of nodes at both start and end of the edge, respectively, $c_{\max\_node}$ is the maximum number of direct references in the network, and $h$ is the weight value that balances direct and indirect references. The value of $h$ was set to 0.85 according to the previously published literature [S5]. The number of direct references of a node is equal to the number of citations of the literature. The Search Path count (SPC) algorithm is used to calculate the indirect references of an edge. The SPC algorithm, as shown in Figs. S1a-b, defines the weight of an edge, which is the number of paths that contain this edge. For example, the weight value of edge C->D is 2 because only the path A->C->D->F->J and A->C->D-H contain the edge C->D.

In the searching step, the search algorithm is employed to get the main path in the citation network. Different search algorithms yield different results and the commonly used search algorithms are shown in Figs. S1c-f. As shown in Fig. S1c, the forward local search algorithm starts from nodes that no nodes point to. Then it selects the edge with the most prominent weight value for each forward walk. It can identify nodes with high citations. The backward local search algorithm, as shown in Fig. S1d, starts from nodes that do not point to any node. Then, it selects the edge with the most prominent weight value for each back walk. It can identify nodes that draw inspiration from multiple previous papers. In Fig. S1e, the global search algorithm selects the path with the highest weight and the most influential path in the graph. The key-route search algorithm selects k edges with the highest weight values from the citation network and then uses the nodes at both ends of the k edges as beginning nodes for forward and backward search. The result is shown in Fig. S1f and it cover edges with the highest weight value in the citation network.


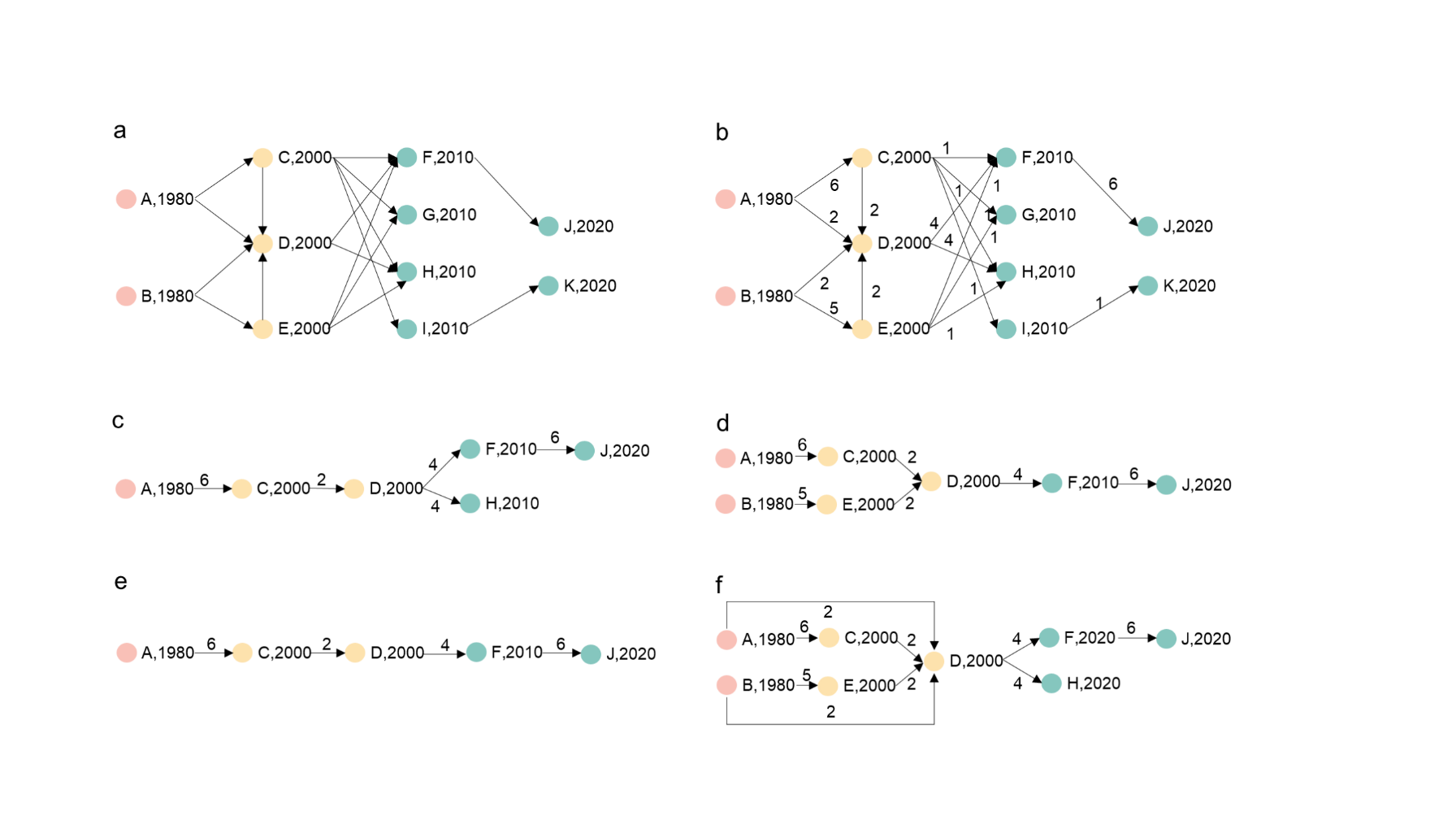


Fig. S1 (**a**) A simple citation network example. (**b**) The weighted citation network is obtained using SPC algorithm, where the weight of an edge is defined as the number of paths containing the edge. (**c**) A forward local main path. The forward local search algorithm is applied in the weighted citation network. (**d**) A backward local main path. The backward local search algorithm is applied in the weighted citation network. (**e**) A global main path. The global search algorithm is applied in the weighted citation network. (**f**) A key-route main path. The key-route search algorithm selects top $k$ ($k=2$) edges from the weighted citation network

## S1.6 Weighted centrality

The main path analysis algorithm mainly concentrates on the propagation path of knowledge among nodes, and the selected nodes mostly play a bridging role in connecting different knowledge domains, but may not be the most influential nodes in that domain. For example, if the predecessor node of a node has a weak influence in the reference network, the node may not be recognized by the main path analysis algorithm, even if it is a high influence node representing technological breakthroughs. To better seek the most influential papers, we calculated the weighted centrality of the nodes in the main path analysis step of our model, as shown in the following formula:

$Centrality(n_{i})=\frac{0.5\sum_{\begin{matrix} j=1 \\ (j\neq i) \end{matrix}}^{n} weight(e_{ji})+0.5\sum_{\begin{matrix} j=1 \\ (j\neq i) \end{matrix}}^{n} weight(e_{ij})}{n-1},$ [S15]

where $weight(e_{ji})$ is the edge weight value from node $j$ to node $i$, and $n$ is the number of nodes in the network. The result is shown in Table S3, which shows the top 50 ranked nodes with their corresponding weighted centrality values. We found that papers with a longer publication time are more likely to be selected because their citation count accumulates year by year. This will make it difficult to discover the latest published valuable papers. Therefore, we eliminate the impact of publication time on the number of citations by calculating the annual average number of citations. The improved formula is as follows:

$weight(e_{i})=h\frac{in_{e_{i}}}{in_{\max\_edge}(2021-t_{p})}+(1-h)\frac{c_{ns}+c_{ne}}{c_{\max\_node}(2021-t_{p})},1980\leq t_{p}\leq2020,$ [S16]

where $t_{p}$ is the publication year and used to address the impact of time on weights, $e_{i}$ refers to an edge and $in_{e_{i}}$ is the number of indirect references of $e_{i}$, $in_{\max\_edge}$ is the maximum number of indirect references in the network, $c_{ns}$ and $c_{ne}$ are the number of direct references of nodes at start and end of the edge, respectively, $c_{\max\_node}$ is the maximum number of direct references in the network, and $h$ is the weight value that balances direct and indirect references. It can be used to predict the most influential papers in the future. We applied it to the citation network from 2015 to 2020 because this period was close and contained sufficient data. The result was shown in Table S4.

## Table S3 Top 50 nodes in weighted centrality ranking

| Node Label | Weighted Centrality |
| --- | --- |
| Ferrari Ac, 2000, Phys. Rev. B | 410.8179480 |
| Nie Sm, 1997, Science | 400.4135509 |
| Kneipp K, 1997, Phys. Rev. Lett. | 210.5562043 |
| Stankovich S, 2007, Carbon | 83.05048924 |
| Ferrari Ac, 2001, Phys. Rev. B | 48.69270643 |
| Pimenta Ma, 2007, Phys. Chem. Chem. Phys. | 44.96815642 |
| Li Jf, 2010, Nature | 41.79541112 |
| Knight Ds, 1989, J. Mater. Res. | 36.83353853 |
| Xu Hx, 1999, Phys. Rev. Lett. | 36.31850787 |
| Cancado Lg, 2011, Nano Lett. | 31.83084024 |
| Le Ru Ec, 2007, J. Phys. Chem. C | 31.42547910 |
| Stiles Pl, 2008, Annu. Rev. Anal. Chem. | 31.34172835 |
| Hildebrandt P, 1984, J. Phys. Chem. | 23.18299703 |
| Ferrari Ac, 2004, Philos. Trans. R. Soc. A-Math. Phys. Eng. Sci. | 23.05670263 |
| Graf D, 2007, Nano Lett. | 23.04623980 |
| Sadezky A, 2005, Carbon | 22.88040607 |
| Kudin Kn, 2008, Nano Lett. | 22.45846418 |
| Defaria Dla, 1997, J. Raman Spectrosc. | 22.36616928 |
| Moskovits M, 2005, J. Raman Spectrosc. | 20.57984196 |
| Xu Hx, 2000, Phys. Rev. E | 18.52583742 |
| Dresselhaus Ms, 2010, Nano Lett. | 16.55673917 |
| Michaels Am, 1999, J. Am. Chem. Soc. | 16.24232629 |
| Rao Am, 1997, Science | 14.63124563 |
| Cançado Lg, 2006, Appl. Phys. Lett. | 14.15728966 |
| Wang Y, 1990, Chem. Mat. | 13.30992284 |
| Hernandez Y, 2008, Nat. Nanotechnol. | 12.23975271 |
| Stöckle Rm, 2000, Chem. Phys. Lett. | 11.16059699 |
| Schwan J, 1996, J. Appl. Phys. | 10.81462822 |
| Kataura H, 1999, Synth. Met. | 10.50650808 |
| Mohiuddin Tmg, 2009, Phys. Rev. B | 10.33192099 |
| Michaels Am, 2000, J. Phys. Chem. B | 9.898260261 |
| Haynes Cl, 2005, Anal. Chem. | 9.137423045 |
| Yang D, 2009, Carbon | 9.038992178 |
| Lee C, 2010, ACS Nano | 9.007118402 |
| De Gelder J, 2007, J. Raman Spectrosc. | 8.974803334 |
| Reich S, 2004, Philos. Trans. R. Soc. A-Math. Phys. Eng. Sci. | 8.594195236 |
| Jiang J, 2003, J. Phys. Chem. B | 8.244244418 |
| Leopold N, 2003, J. Phys. Chem. B | 8.018884784 |
| Eckmann A, 2012, Nano Lett. | 7.977934755 |
| Lucchese Mm, 2010, Carbon | 7.709142898 |
| Jorio A, 2001, Phys. Rev. Lett. | 7.273995339 |
| Mcfarland Ad, 2005, J. Phys. Chem. B | 7.058803209 |
| Zhang R, 2013, Nature | 7.045548484 |
| Fang Y, 2008, Science | 7.030774363 |
| Noda I, 1993, Appl. Spectrosc. | 6.894838552 |
| Moskovits M, 1984, J. Phys. Chem. | 6.747340379 |
| Lieber Ca, 2003, Appl. Spectrosc. | 6.570009792 |
| Hao E, 2004, J. Chem. Phys. | 6.562370059 |
| Bachilo Sm, 2002, Science | 6.517653431 |
| Li H, 2012, Adv. Funct. Mater. | 5.999021528 |

**Table S4** Top 30 nodes predicted to have greater impact in the future

| Node Label | Weighted Centrality |
| --- | --- |
| Butler Hj, 2016, Nat. Protoc. | 5.285162 |
| Schultz Jf, 2020, Appl. Spectrosc. | 4.075938 |
| Xu Kc, 2019, Adv. Sci. | 3.384142 |
| Sartin Mm, 2020, J. Chem. Phys. | 3.045923 |
| Zhou X, 2020, Adv. Sci. | 2.905840 |
| Yang Sk, 2016, Proc. Natl. Acad. Sci. U. S. A. | 2.447562 |
| Shi Ry, 2018, J. Agric. Food Chem. | 2.323390 |
| Xu Lj, 2015, J. Am. Chem. Soc. | 1.778314 |
| Zheng Xs, 2018, Spectroc. Acta Pt. A-Molec. Biomolec. Spectr. | 1.593270 |
| Neumann C, 2015, Nat. Commun. | 1.526427 |
| Lee J, 2019, Nature | 1.426108 |
| Mahapatra S, 2020, J. Chem. Phys. | 1.276714 |
| Chen Jm, 2016, Anal. Chem. | 1.261652 |
| Shen W, 2015, Angew. Chem.-Int. Edit. | 1.187938 |
| Zhong Jh, 2017, Nat. Nanotechnol. | 1.177597 |
| Chen Hy, 2015, J. Am. Chem. Soc. | 1.169970 |
| Liu K, 2016, Nano Lett. | 1.161217 |
| Chiang Nh, 2016, Nano Lett. | 1.149531 |
| Deng Yl, 2016, ACS Catal. | 1.144889 |
| Joseph Mm, 2018, Biomaterials | 1.133486 |
| Jermyn M, 2015, Sci. Transl. Med. | 1.126298 |
| De Jesus Jisd, 2020, J. Pharm. Pharm. Sci. | 1.078936 |
| Neng J, 2020, Biosens. Bioelectron. | 1.072802 |
| Panneerselvam R, 2018, Chem. Commun. | 1.066623 |
| Cong S, 2015, Nat. Commun. | 1.035766 |
| Mignuzzi S, 2015, Phys. Rev. B | 0.987622 |
| Zhu Ch, 2016, Adv. Mater. | 0.973914 |
| Benz F, 2016, Science | 0.963786 |
| Jiang S, 2015, Nat. Nanotechnol. | 0.920982 |
| Li Cy, 2020, Nat. Nanotechnol. | 0.908234 |

# S2 Results of Ablation and Empirical Study

## S2.1 Ablation study

The BERTopic model provides a modular framework in which the dimensionality reduction and clustering modules have an impact on the results of topic extraction. So, we tried several conventional dimensionality reduction algorithms and clustering algorithms and planned to select the best combination that is most suitable for Raman spectroscopy. The t-Distributed Stochastic Neighbor Embedding (t-SNE) and UMAP algorithms are two traditional dimensionality reduction algorithms that can preserve the structural information of the data well. The Hierarchical Density-Based Spatial Clustering of Applications with Noise (HDBSCAN), Agglomerative Clustering, and Balanced Iterative Reducing and Clustering using Hierarchies (BIRCH) algorithms are three classical unsupervised clustering algorithms that can handle clusters of different shapes and sizes. We combined the above algorithms and ran them on our dataset, and the results are shown in Table S5. It can be observed that the model performs best when the dimensionality reduction algorithm is UMAP and the clustering algorithm is HDBSCAN.

## Table S5 BERTopic model performance under different combinations of dimensionality reduction and clustering algorithms

| Model | NPMI | Diversity |
| --- | --- | --- |
| t-SNE_HDBSCAN | 0.02 | 0.77 |
| t-SNE_AgglomerativeClustering | 0.02 | 0.86 |
| t-SNE_BIRCH | 0.02 | 0.68 |
| UMAP_HDBSCAN | 0.08 | 0.86 |
| UMAP_AgglomerativeClustering | 0.01 | 0.75 |
| UMAP_BIRCH | -0.20 | 1.00 |

## S2.2 Determination of the threshold for topic evolution

To obtain information about topic evolution in the development process of Raman spectroscopy, the semantic similarity among topics was measured by the Cosine similarity method to establish the evolutionary relationship among them. It is well-accepted that an evolutionary relationship exists between two topics only when the similarity is more significant than a certain threshold. However, it is a problem to get an appropriate value. Finally, we decided to combine the enumeration method with specialized knowledge to get the threshold.

Here, we assumed that the threshold was $\varepsilon\cdot max(\varepsilon\in(0,1))$, where $max$ is the maximum similarity value. Figs. S2-S6 show the topic evolution results between the emerging (T_n_) and growth (T_n+1_) stages, where $\varepsilon$ varies within the range between 0.5 and 0.9 with a step size of 0.1. When the $\varepsilon$ is 0.5 or 0.6, all topics in the emerging stage are almost merged into topics in the growth stage (Figs. S2 and S3). Topic evolution is a complex process influenced by many factors. Sometimes, a topic disappears due to its low research value and insufficient technological level at the time. It is abnormal that more than 25% of the topics in the emerging stage have evolutionary relationships with about 75% of the topics in the growth stage. Hence, we thought that the situation in the figure did not conform to realistic logic, and the $\varepsilon$ was greater than 0.6.


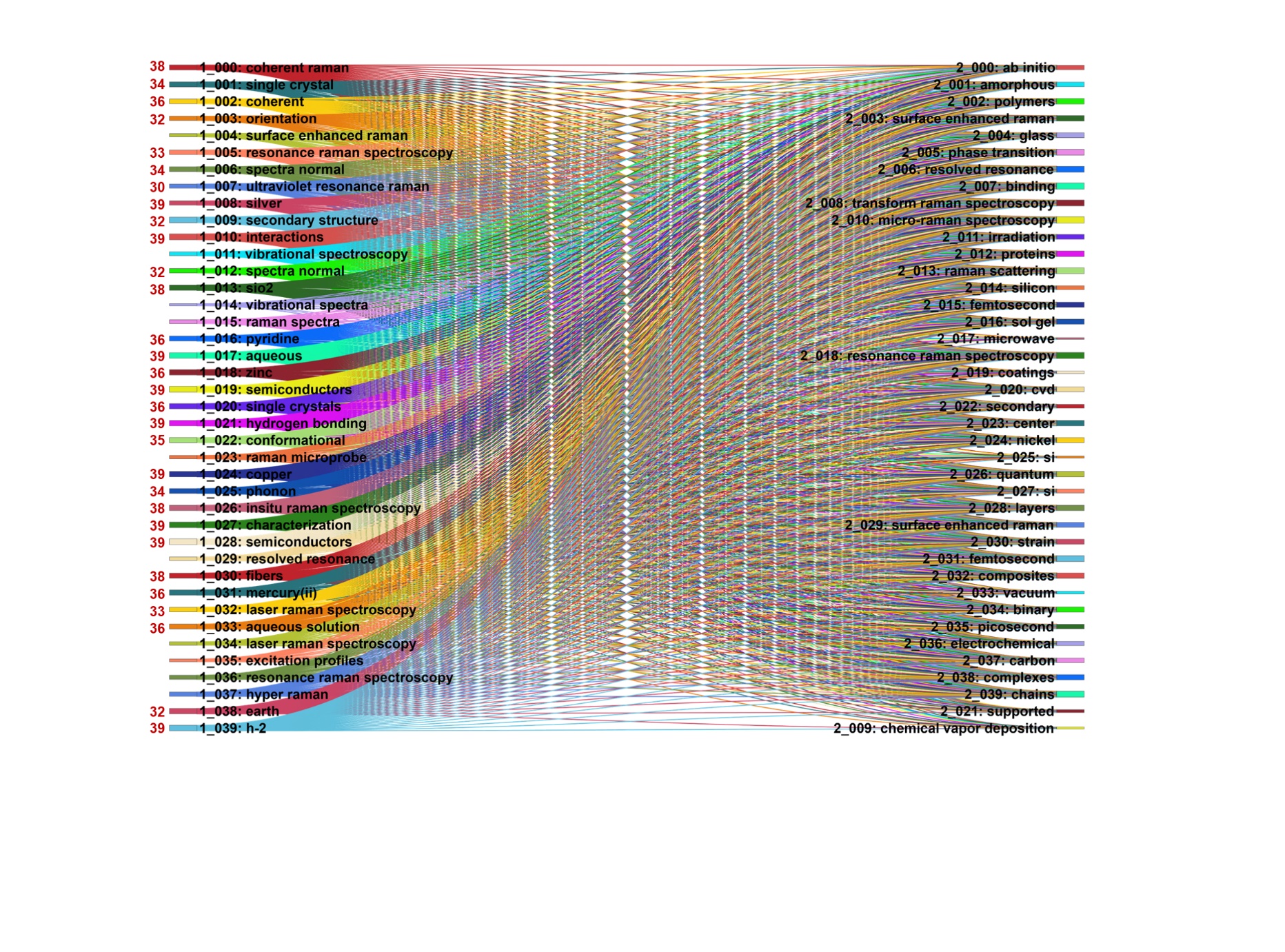


## Fig. S2 Sankey diagram showing the topic evolution relationship between the emerging and growth stage when $\varepsilon$ is 0.5. Each topic is represented by its stage, topic number, and the first keyword. The number 1 and 2 before the underline indicates that the topic is in the emerging and growth stage, respectively. The links derived from each topic is the same as the color of the node itself. There are 40 topics in the emerging and growth stage that have evolutionary relationships. Topics with 30 or more links in the emerging stage are colored in red.

**
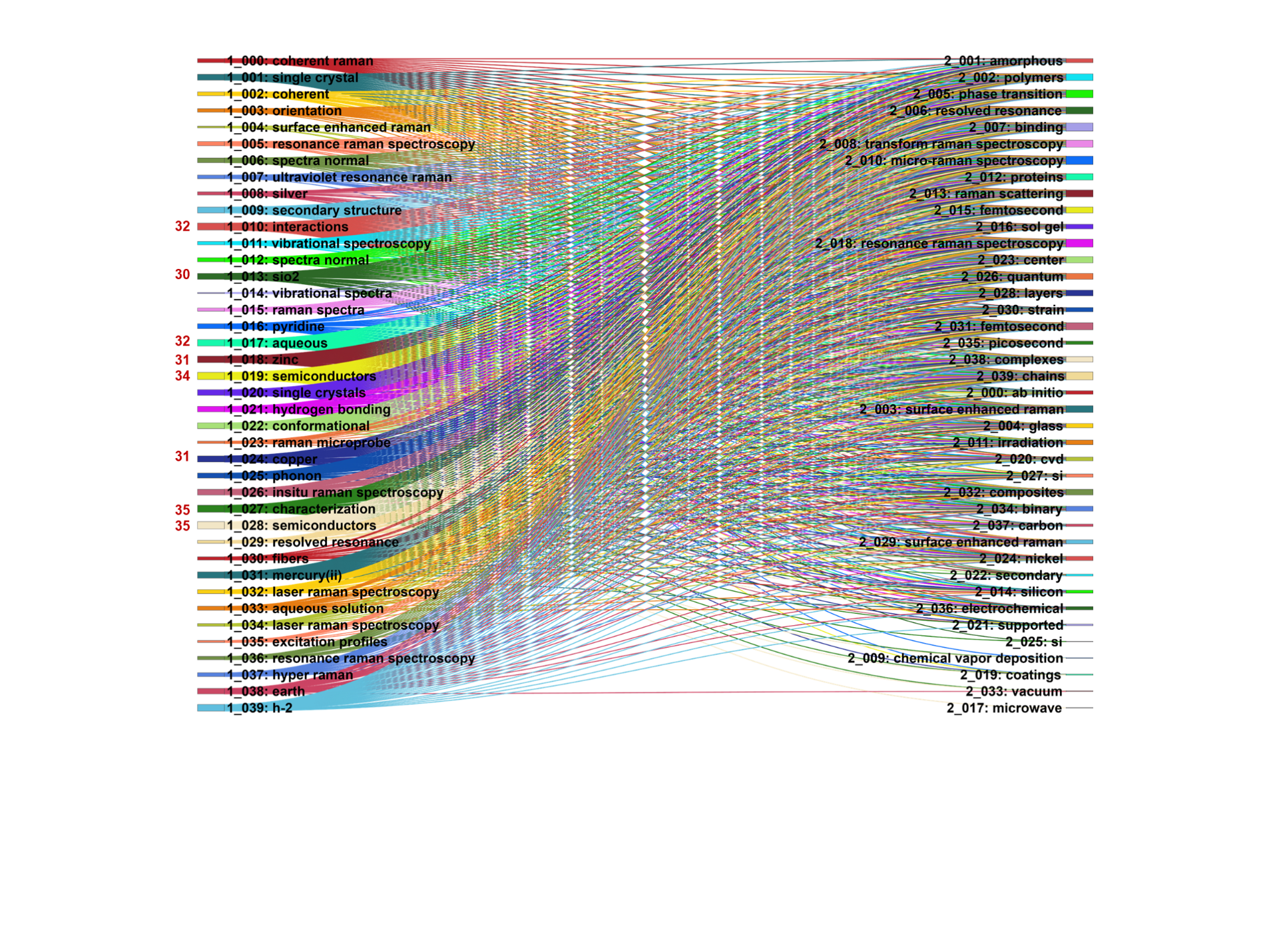
**

## Fig. S3 Sankey diagram showing the topic evolution relationship between the emerging and growth stage when $\varepsilon$ is 0.6. Each node is represented by its stage, topic number, and the first keyword. The number 1 and 2 before the underline indicates that the topic is in the emerging and growth stage, respectively. The links derived from each node is the same as the color of the node itself. There are 40 topics in the emerging and growth stage that have evolutionary relationships. Topics with 30 or more evolutionary paths in the emerging stage are colored in red.

When the $\varepsilon$ is 0.7, the topic evolution is more logical compared to previous results (Fig. S4). But we found some wrong evolutionary relationships in the graph. For example, the topic terms for topic 1_008 are silver, surface enhanced Raman, enhanced Raman spectroscopy, scattering, deposited, surface enhanced resonance, adsorption, selection rules, acid, and resonance Raman scattering. The topic terms for topic 2_024 are nickel, electrochemical, oxide, spectroscopy study, in-situ, layer, identification, Raman spectroscopy study, surface enhanced Raman, and film. We thought that topic 1_008 described adsorption analysis on silver surfaces using Raman spectroscopy, while topic 2_024 involved electrochemical oxidation of nickel materials, including in-situ spectroscopy analysis and layer identification. There was no strong correlation between them. In addition, we also used the Jaccard Index for verification. The result indicated a weak correlation. When the $\varepsilon$ is 0.9, there are too few topics with evolutionary relationships (Fig. S6). Above all, we thought the appropriate threshold is 0.8 (Fig. S5).


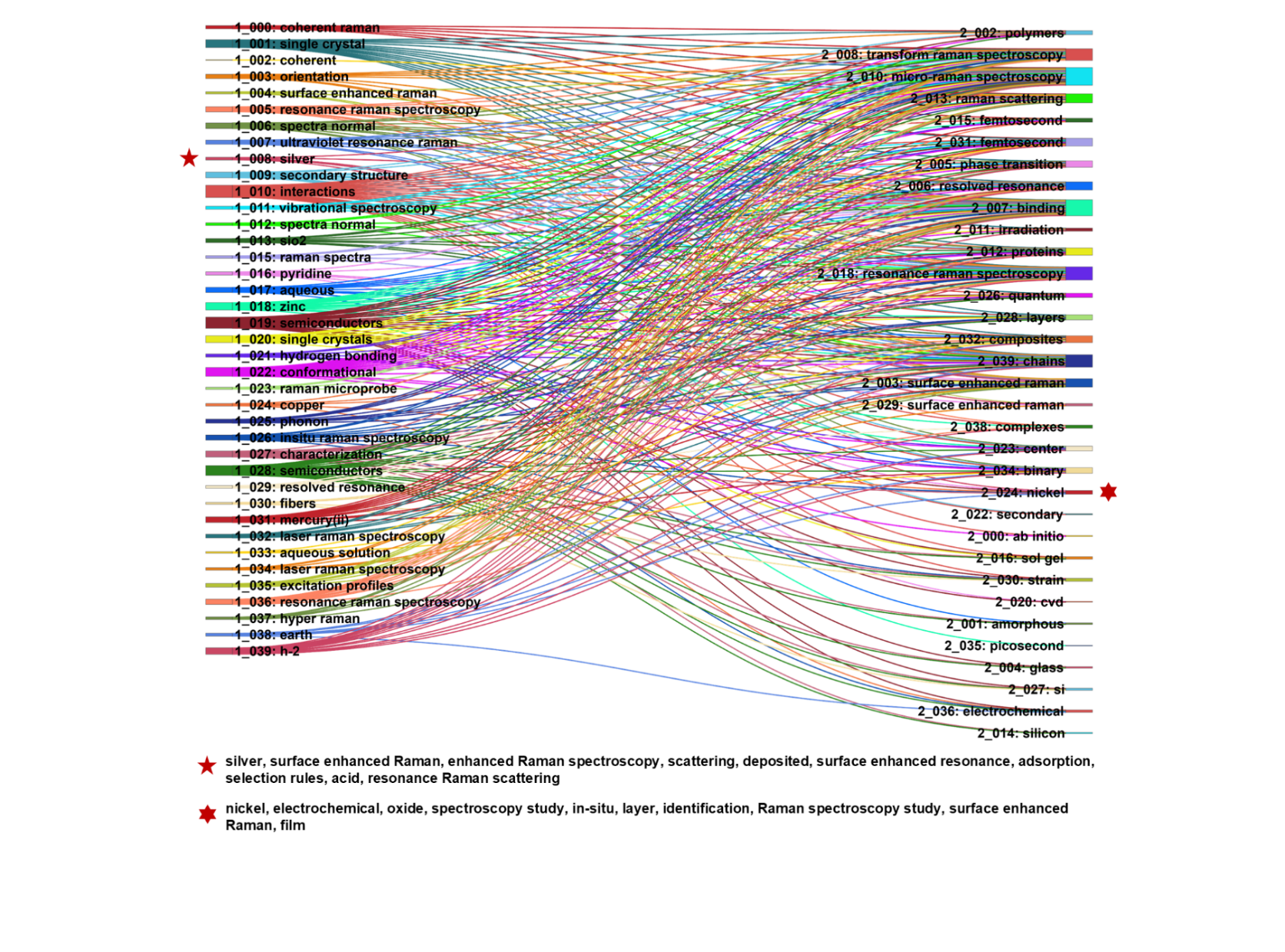


## Fig. S4 Sankey diagram showing the topic evolution relationship between the emerging and growth stage when $\varepsilon$ is 0.7. Each node is represented by its stage, topic number, and the first keyword. The number 1 and 2 before the underline indicates that the topic is in the emerging and growth stage, respectively. The links derived from each node is the same as the color of the node itself. Because the threshold is not suitable, there are some wrong evolutionary relationships in the diagram, such as the evolutionary relationship marked by red stars. The words next to the red star are the topic words corresponding to the two topics.


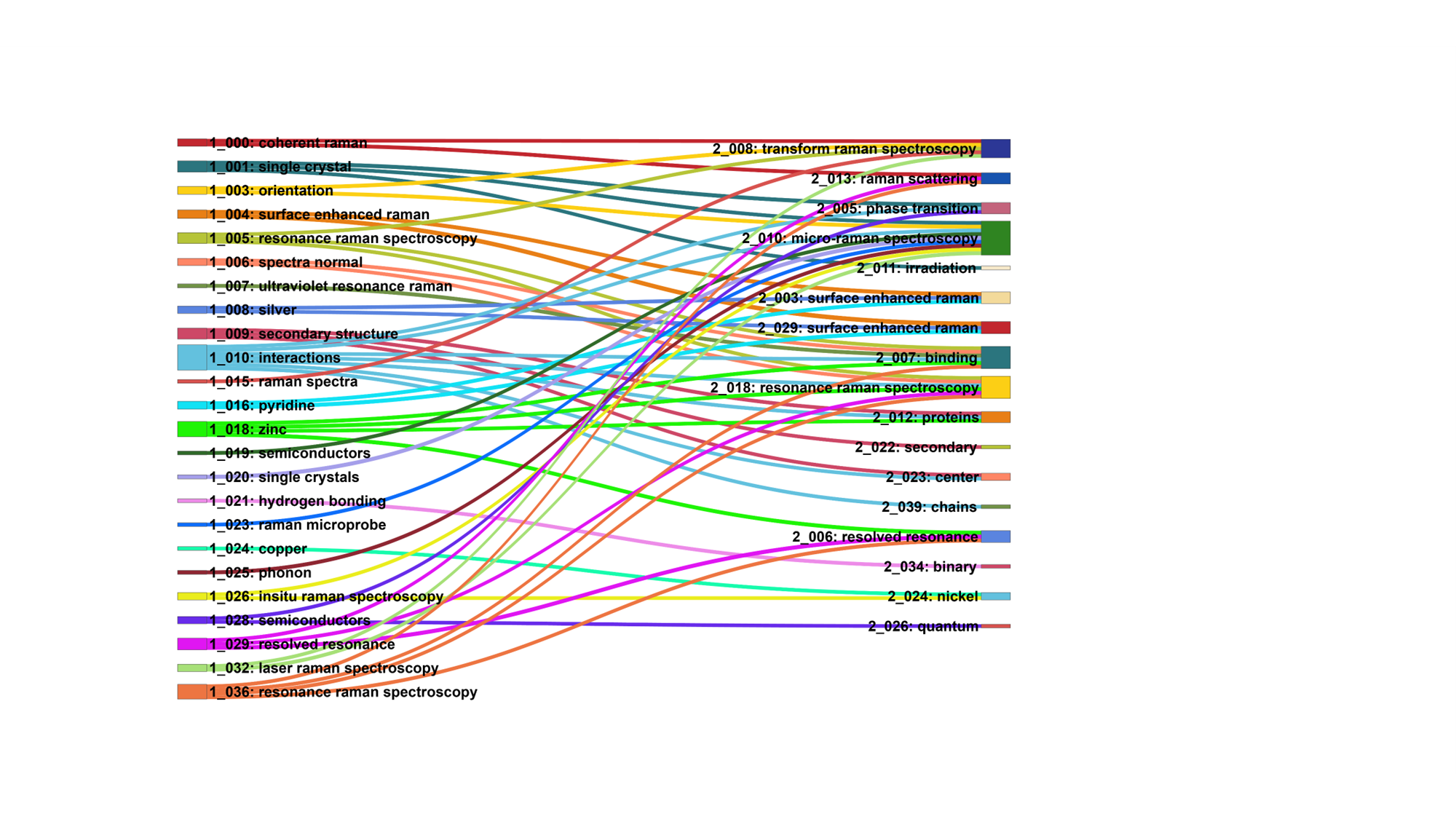


## Fig. S5 A Sankey diagram showing the topic evolution relationship between the emerging and growth stage when $\varepsilon$ is 0.8. Each node is represented by its stage, topic number, and the first keyword. The number 1 and 2 before the underline indicates that the topic is in the emerging and growth stage, respectively. The link derived from each node is the same color as the node.


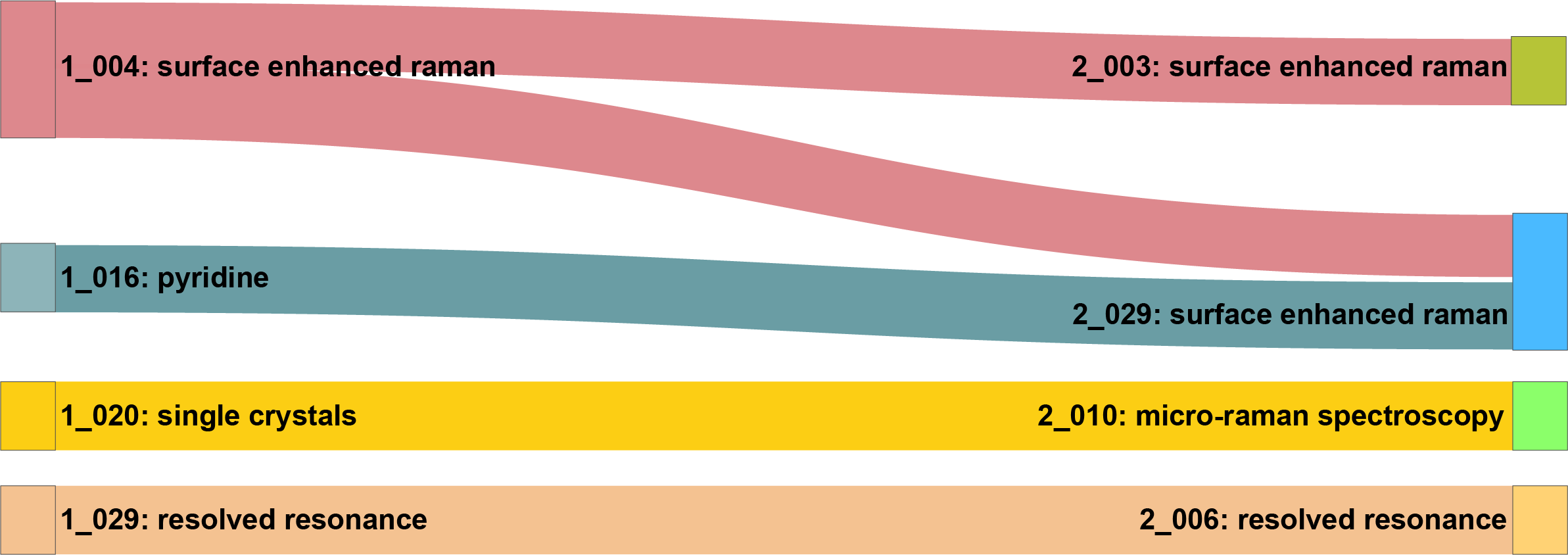


## Fig. S6 A Sankey diagram showing the topic evolution relationship between the emerging and growth stage while $\varepsilon$ is 0.9. Each node is represented by its stage, topic number, and the first keyword. The number 1 and 2 before the underline indicates that the topic is in the emerging and growth stage, respectively. The link derived from each node is the same color as the node.

The topic evolution relationship between the growth and maturity stages is determined in the same way, and the $\varepsilon$ varies within the range between 0.85 and 0.90 with a step size of 0.01. We ultimately considered the threshold of 0.88 to be the most suitable. Finally, we obtained all the evolution relationships, as shown in Fig. S7.


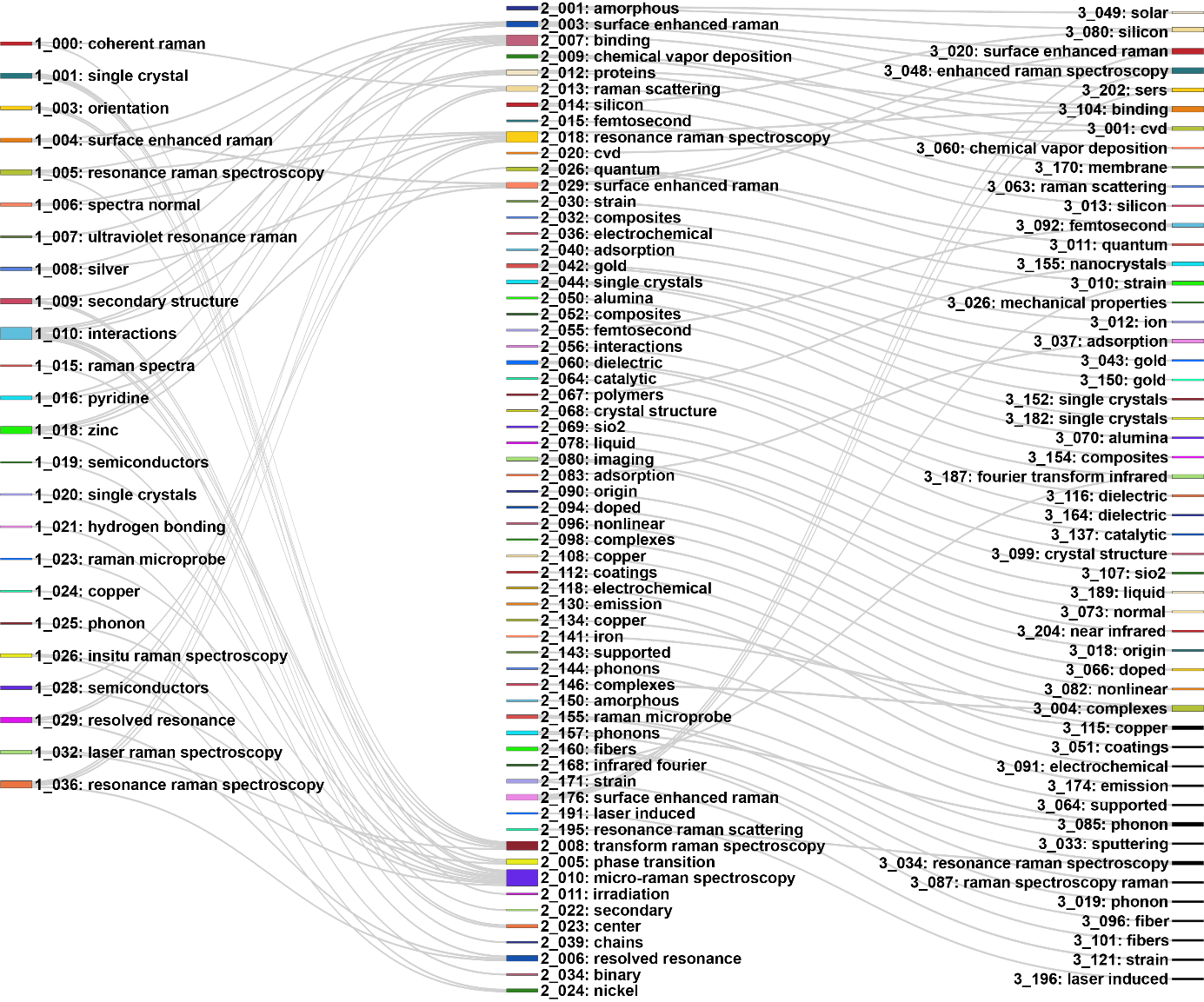


## Fig. S7 The complete result of topic evolution. Each node is represented by its stage, topic number, and the first keyword. The number 1 and 2 before the underline indicates that the topic is in the emerging and growth stage, respectively.

## S2.3 Analysis of the forward main path

The forward local search algorithm was used to simplify the weighted citation network graph and obtain the forward main path graph, as shown in Fig. S8. As we can see, the edge from node ‘Hildebrandt P, 1984, J. Phys. Chem.’ to node ‘Nie Sm, 1997, Science’ has the highest weight value, and node ‘Nie Sm, 1997, Science’ has the highest citation count. So, we thought that the most important node was the paper published in Science in 1997. This paper validated the capability of surface-enhanced Raman spectroscopy to detect a single molecule adsorbed on an individual nanoparticle at room temperature for the first time [S6]. It is considered as the origin of single-molecule Raman spectroscopy and a significant milestone paper in the history of Raman spectroscopy. It proves that our algorithm can accurately capture important research results in the academic field.


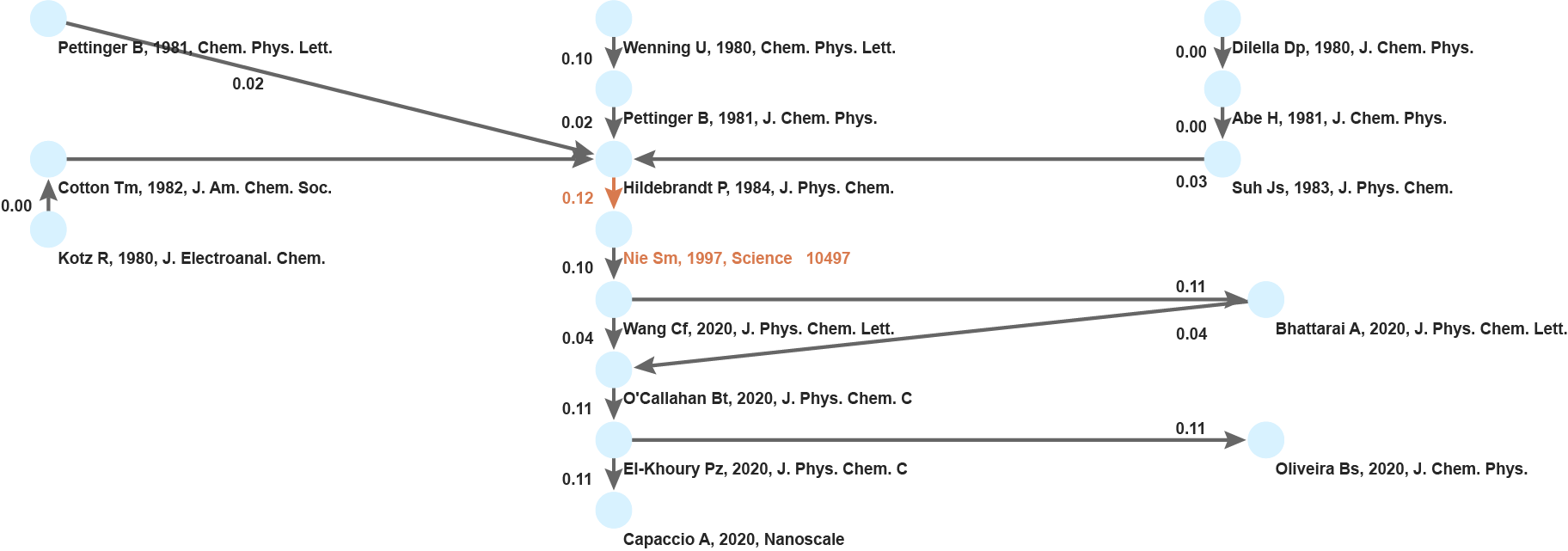


## Fig. S8 The forward local main path of Raman spectroscopy. The label of a node consists of the name of the first author, publication year, and journal abbreviation. The edges are marked with their corresponding weight values. The orange edge is the one with the highest weight value in the diagram. The label of the node marked in orange is the one with the highest citation count in the diagram and the last number is citation count of the node.

## S2.4 Analysis of the backward main path

The backward local search algorithm was used to simplify the weighted citation network and obtain the backward main path graph, and the result is shown in Fig. S9. It can be observed that the paper published by E. C. Le Ru et al. in 2007 has the highest citation count. They deeply studied the enhancement factor (EF) and cross section of surface enhanced Raman scattering (SERS) and found that when SERS EF was as low as 10^7^, it was sufficient to observe single molecule SERS signals [S7]. The edge with the highest weight value in Fig. S9 is the edge from node ‘Mrozek Mf, 2002, Anal. Chem.’ to node ‘Shafer-Peltier Ke, 2003, J. Am. Chem. Soc.’. The previous node demonstrated the detection and identification of aqueous saccharides through SERS [S8]. The latter node reported the direct detection of glucose by using SERS, which provided the possibility for the application of SERS in the glucose biosensors [S9].

**
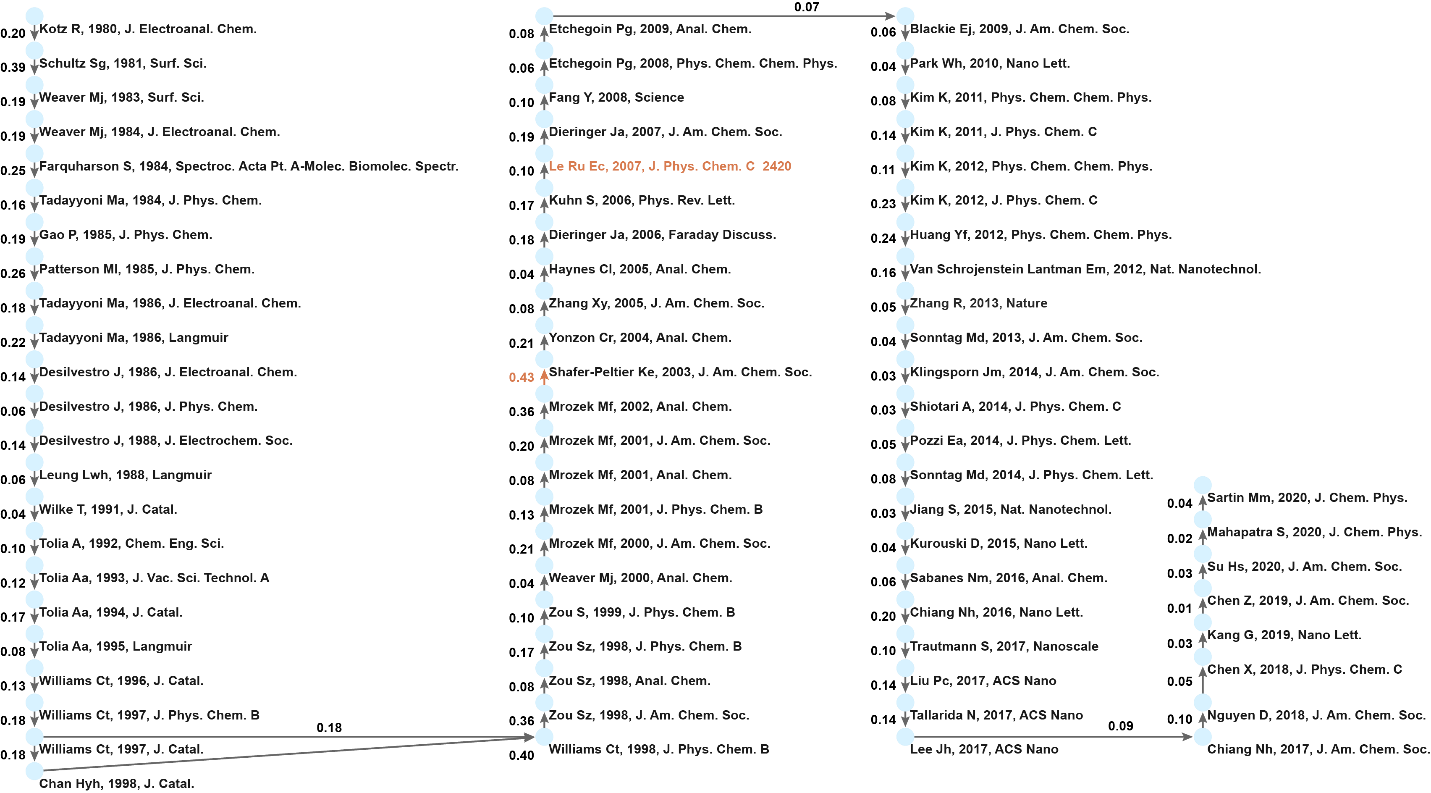
**

## Fig. S9 The backward local main path of Raman spectroscopy. The label of a node consists of the name of the first author, publication year, and journal abbreviation. The edges are marked with their corresponding weight values. The orange edge is the edge with the highest weight value in the diagram. The label of the node marked in orange is the one with the highest citation count in the diagram and the last number is citation count of the node.

## S2.5 Analysis of the global main path

The global main path is shown in Fig. S10 and the node with the highest citation count is the same as the node of the backward main path. The edge with the highest weight value in Fig. S10 is the edge from node ‘Chan Hyh, 1999, J. Phys. Chem. B’ to node ‘Zou Sz, 1999, J. Phys. Chem. B’. The previous node explored the electrode potential-dependent formation of oxygen species on copper in noncomplexing aqueous media at different pH values utilizing SERS [S10]. The latter node demonstrated the characterization of phonon and related interfacial vibrations within ultrathin semiconductor layers on gold substrates by using SERS [S11].


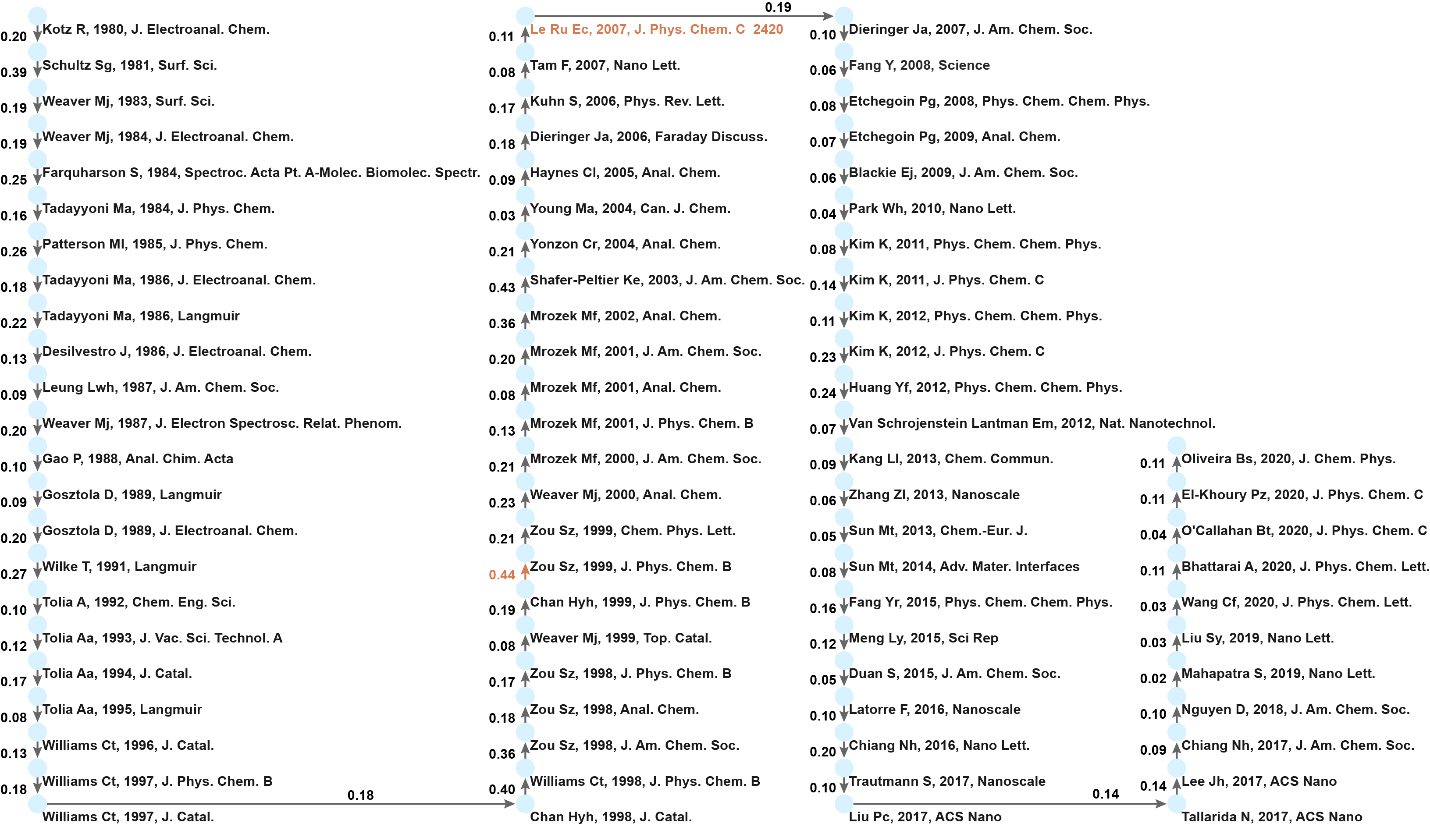


## Fig. S10 The global main path of Raman spectroscopy. The label of a node consists of the name of the first author, publication year, and journal abbreviation. The edges are marked with their corresponding weight values. The orange edge is the edge with the highest weight value in the diagram. The label of the node marked in orange is the one with the highest citation count in the diagram and the last number is citation count of the node.

## S2.6 Analysis of key-route main path

The key-route main path, as shown in Fig. S11, contains 94 nodes, which has the maximum number of nodes in all main path graphs. Starting from node ‘Weaver MJ, 2000, Nanal. Chem.’, every subsequent node has already appeared in the backward main path graph. The node with the highest citation count and the edge with the highest weight value in the key main path graph are the same as those in the global main path graph.


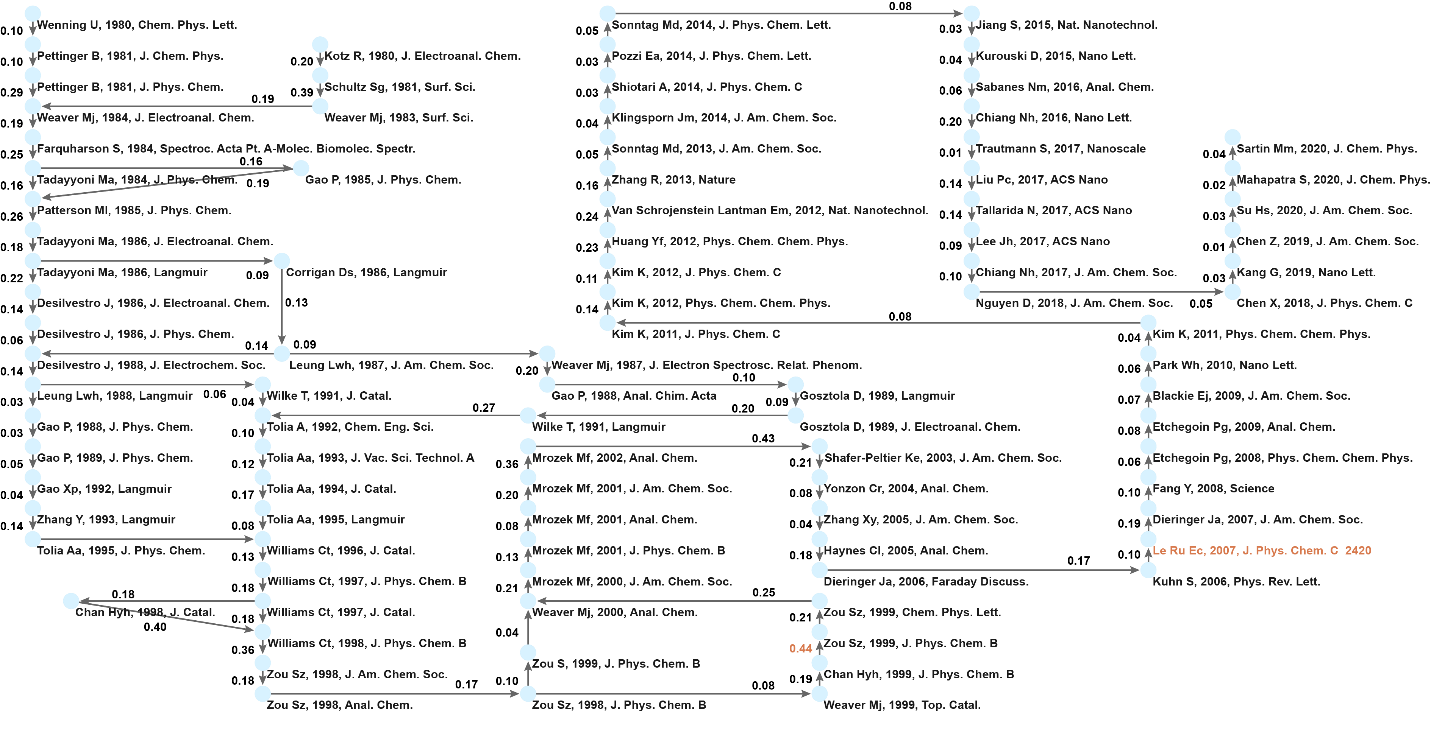


## Fig. S11 The key-route main path of Raman spectroscopy. The label of a node consists of the name of the first author, publication year, and journal abbreviation. The edges are marked with their corresponding weight values. The orange edge is the edge with the highest weight value in the diagram. The label of the node marked in orange is the one with the highest citation count in the diagram and the last number is citation count of the node.

# S3 Supplementary Tables for Topic Model and Citation Analysis Method

## Table S6 Density coefficient of the communities in different periods

| Community | The T_n_ stage | The T_n+1_ stage | The T_n+2_ stage |
| --- | --- | --- | --- |
| Optics | 0.00 | 2.39 | 2.85 |
| Physics | 1.88 | 14.29 | 6.92 |
| Chemistry | 2.16 | 22.70 | 21.39 |
| Spectroscopy | 5.69 | 8.45 | 2.42 |
| Materials Science | 0.00 | 2.29 | 6.10 |
| Biochemistry and Molecular Biology | 0.67 | 1.03 | 0.00 |
| Biophysics | 0.00 | 0.53 | 1.00 |
| Engineering | 0.00 | 0.20 | 0.47 |
| Mineralogy | 0.00 | 0.00 | 1.00 |
| Toxicology | 0.00 | 0.00 | 0.83 |
| Polymer Science | 0.00 | 2.07 | 0.27 |
| Instruments and Instrumentation | 0.00 | 1.00 | 0.83 |
| Astronomy and Astrophysics | 0.00 | 0.25 | 0.40 |
| Geochemistry and Geophysics | 0.00 | 0.83 | 0.00 |
| Meteorology and Atmospheric Sciences | 0.00 | 0.00 | 0.30 |

## Table S7 Topic terms corresponding to the topic

| Topic | Topic words |
| --- | --- |
| 1_004 | surface enhanced Raman, surface enhanced, enhanced Raman, gold, Raman spectroscopy surface, electrochemical, adsorption, probe, interface, scattering |
| 1_008 | silver, surface enhanced Raman, enhanced Raman spectroscopy, scattering, deposited, surface enhanced resonance, adsorption, selection rules, acid, resonance Raman scattering |
| 1_010 | interactions, spectroscopic, Raman spectroscopy study, fluorescence, determined Raman, stretching, order, conformation, proteins, phase transition |
| 1_016 | pyridine, enhanced Raman, surface enhanced, silver electrode, adsorbed, Raman spectroscopy surface, Cu, deposited, transition metal, investigation |
| 1_018 | zinc, ligand, resonance Raman spectroscopy, Raman difference spectroscopy, difference, absorption, binding, studied resonance, picosecond, infrared spectroscopy |
| 1_026 | in-situ Raman spectroscopy, films, electrochemical, copper, using Raman, infrared spectroscopy, use, Raman spectroscopy study, x-ray photoelectron spectroscopy, direct observation |
| 1_029 | red, resolved resonance, resonance Raman spectroscopy, time resolved, resolved resonance Raman, substituted, wavelength, excited states, bacterial, orientational |
| 1_036 | bacterial, resonance Raman spectroscopy, time resolved resonance, picosecond, structural changes, spectroscopy structure, Raman spectroscopy time, determined Raman, flow, binding |
| 2_003 | surface enhanced Raman, adsorbed, SERS, enhanced Raman spectroscopy, scattering study, acid, near infrared, resonance Raman scattering, photochemical, nanoparticles |
| 2_007 | binding, resonance Raman, resonance, ultraviolet, surface enhanced, enhanced Raman spectroscopy, protein, surface enhanced Raman, Raman spectroscopy Raman, double |
| 2_010 | phonon, micro-Raman spectroscopy, films Raman, single crystals, doped, laser, local, spectroscopy Raman, growth, orientation |
| 2_018 | protein, resonance Raman spectroscopy, PH, binding, exchange, complex, transform infrared, ftir, resolved resonance Raman, time resolved resonance |
| 2_024 | nickel, electrochemical, oxide, spectroscopy study, in-situ, layer, identification, Raman spectroscopy study, surface enhanced Raman, film |
| 2_029 | surface enhanced Raman, surface enhanced, SERS, adsorption, charge transfer, probe, substrates, Raman scattering spectroscopy, polymer, Raman |
| 2_176 | cell, surface enhanced Raman, spectroscopy surface, vis, spectroscopy Raman spectroscopy, coated, range, scattering spectroscopy, enhancement, luminescence |
| 3_020 | substrate, surface enhanced Raman, enhanced Raman scattering, enhancement, Raman spectroscopy, hot, spectroscopy, arrays, large scale, nanostructures |
| 3_048 | metallic, enhanced Raman spectroscopy, surface enhanced Raman, Raman scattering, surface, scattering, enhancement, molecule, gap, arrays |
| 3_104 | binding, resonance Raman spectroscopy, time resolved, protein, relaxation, UV, kinetics, sulfide, bonding, spin |
| 3_202 | SERS, array, surface enhanced Raman, surface enhanced, Ag, sensor, enhanced Raman spectroscopy, spectrometry, Raman scattering, scattering |

# S4 Supplementary Figures for Output of Topic Model and Citation Analysis Method


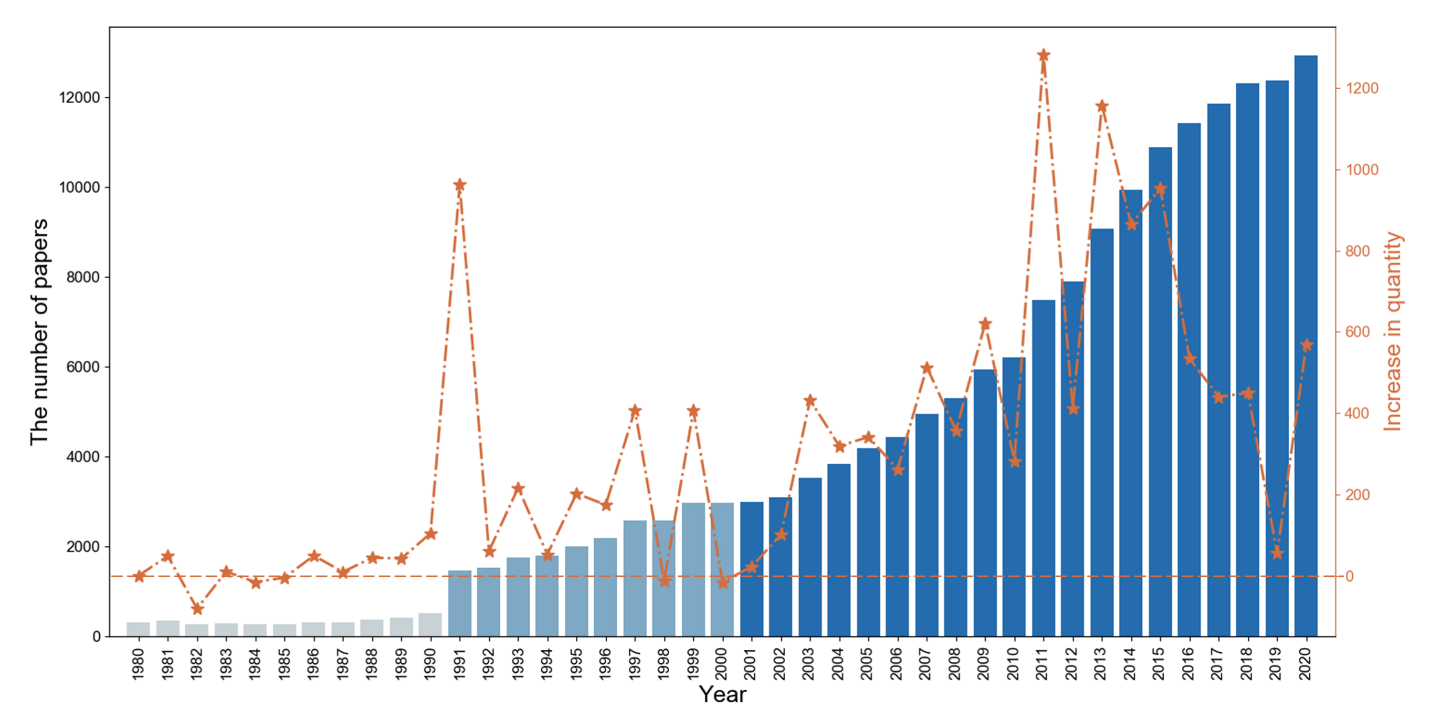


## **Fig. S12** Life cycle diagram of Raman spectroscopy. The bar chart shows the number of publications per year. The orange line represents the yearly increase in publications. According to the number of publications and growth pattern, the period from 1980 to 2020 is divided into three stages: T_n_ stage (emerging stage, from 1980 to 1989), T_n+1_ stage (growth stage, from 1990 to 2000) and T_n+2_ stage (maturity stage, from 2001 to 2020). In the T_n_ stage, the number of publications is less than 500 per year. In the T_n+1_ stage, the number of publications is more than 500 publications per year, and the first-order derivatives greater than 0 are all greater than 50. In the T_n+2_ stage, the first-order derivatives of publications are all greater than 0


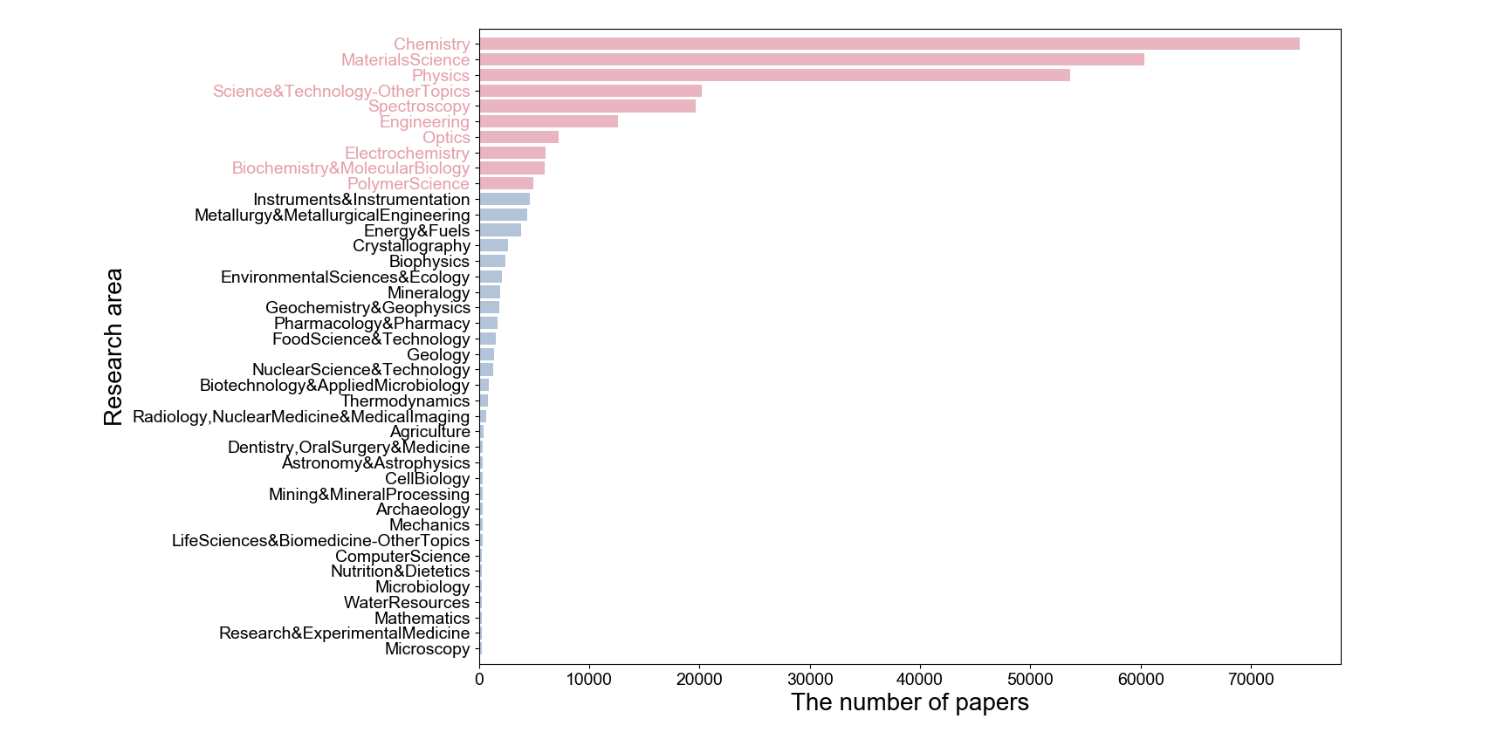


## **Fig. S13** Diagram of the top 40 research areas in terms of the literature amount, with the top 10 highlighted in pink


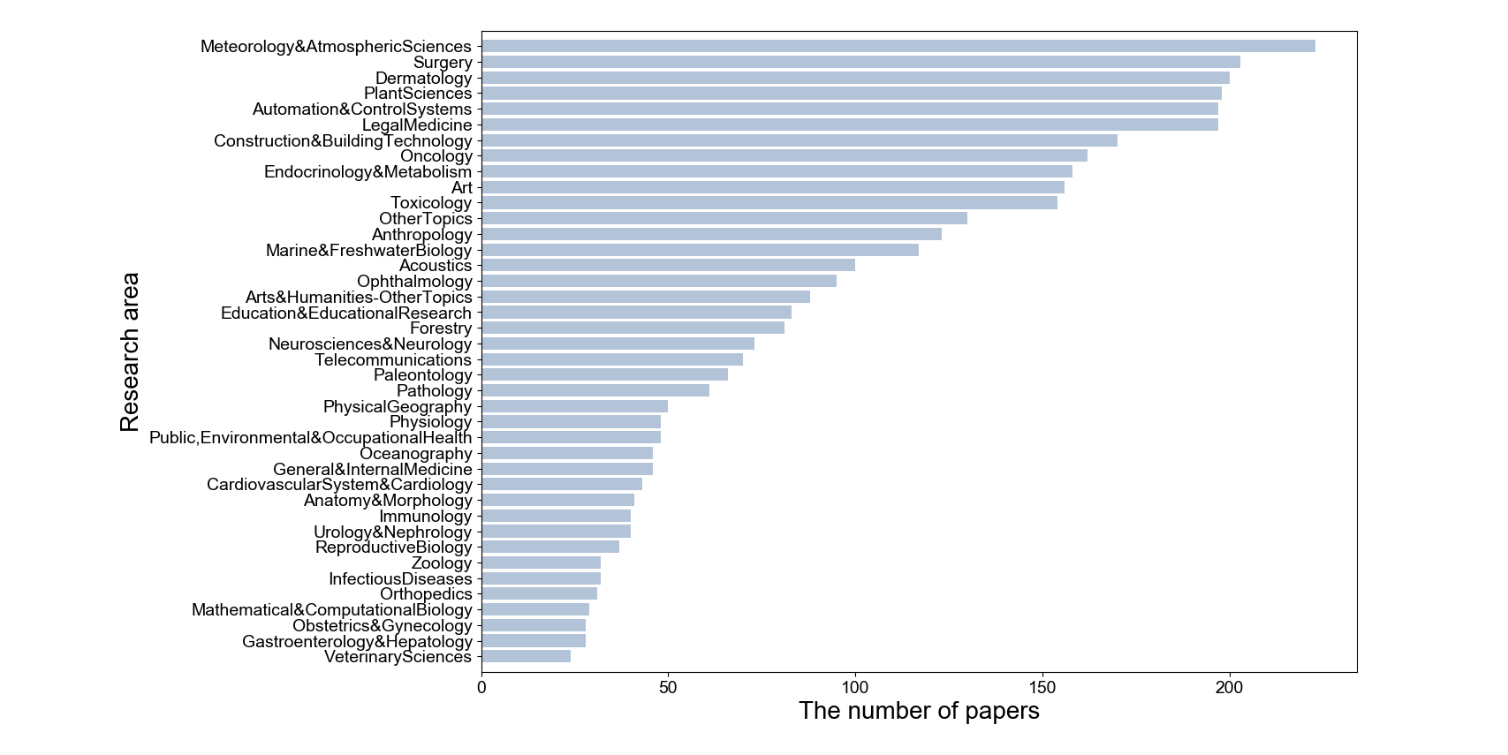


## **Fig. S14** Distribution of research areas ranked 41-80 in terms of literature amount


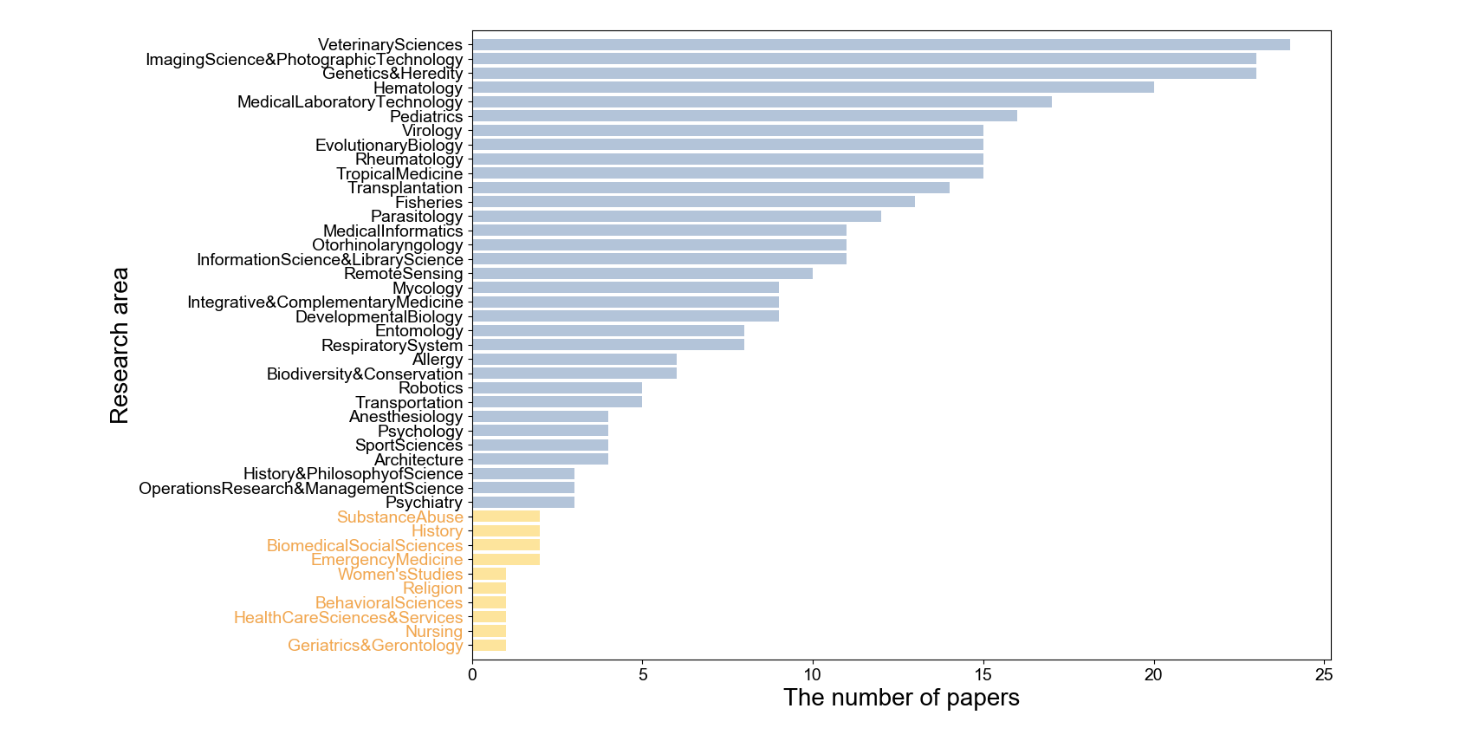


## **Fig. S15** Distribution of research areas ranked 81-122 in terms of literature amount, with the last 10 highlighted in yellow


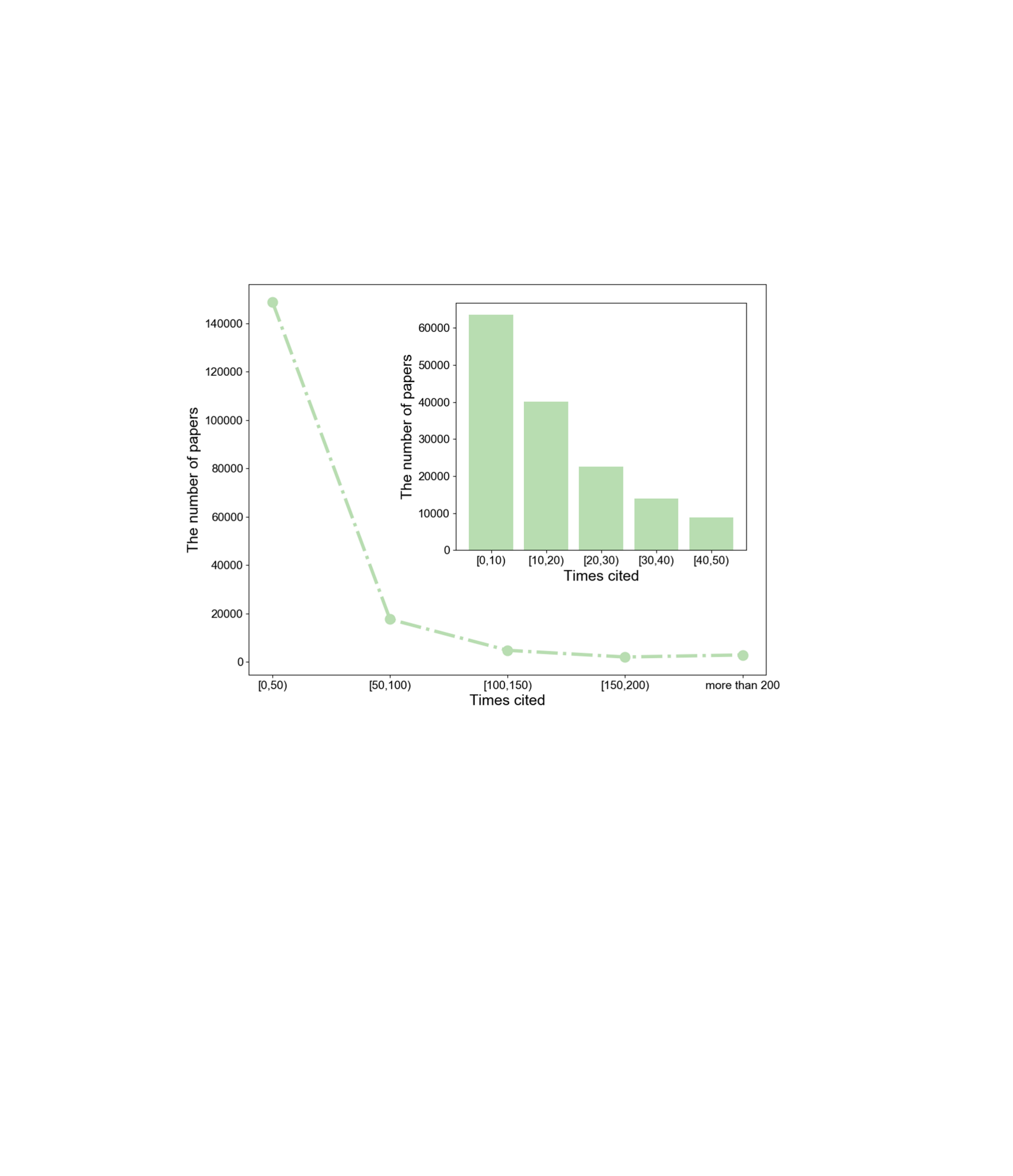


## **Fig. S16** Distribution of citations to literature. The inset shows the detailed distribution of citations to literature between 0 and 50 times

We visualized the similarity of all topics in different stages in Fig. 2. As shown in Figs. S17 and S18, the similarity of most topics between stages T_n+1_ (T2) and T_n+2_ (T3) was greater than that between stages T_n_ (T1) and T_n+1_ (T2).


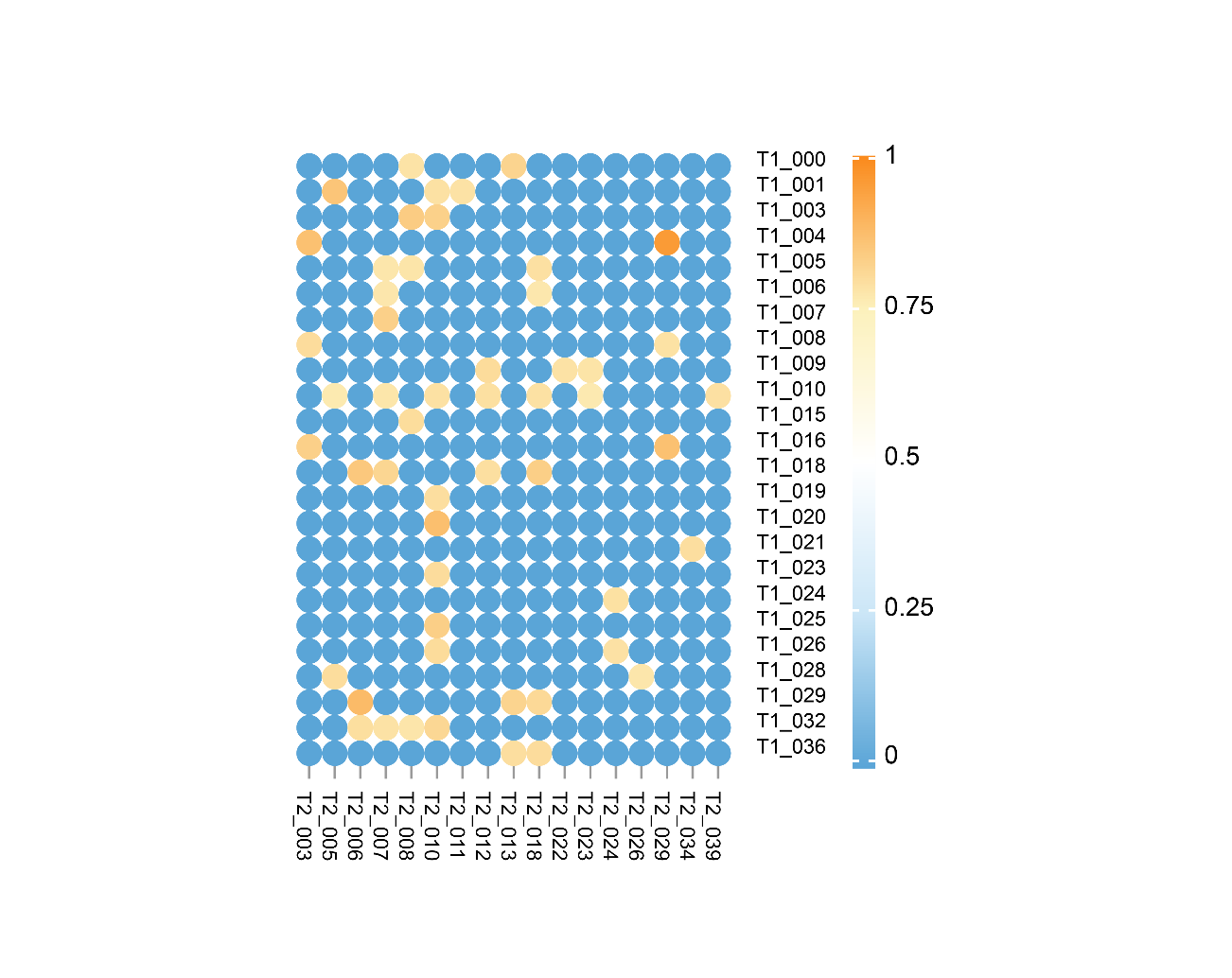


## **Fig. S17** Correlation heatmap of topics between stages T_n_ and T_n+1_. The T1 and T2 represent the stages T_n_ and T_n+1_, respectively


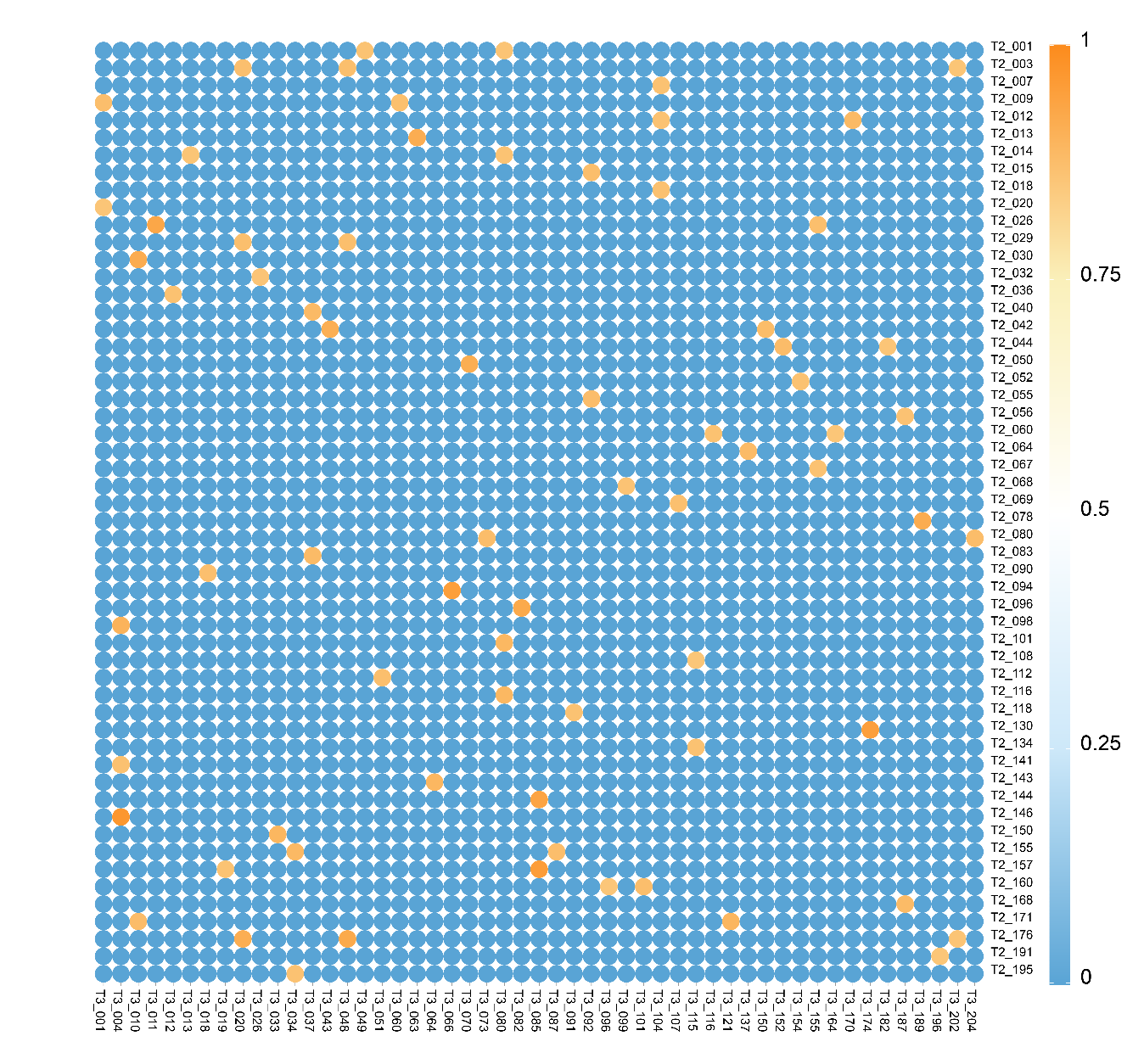


Fig. S18 Correlation heatmap of topics between stages T_n+1_ and T_n+2_. The T2 and T3 represent the stages T_n+1_ and T_n+2_, respectively

# Supplementary References

1. J. Healy, L. McInnes, Uniform manifold approximation and projection. Nat. Rev. Meth. Primers **4**, 82 (2024). <https://doi.org/10.1038/s43586-024-00363-x>
2. D. Bhandari, N.R. Pal, Some new information measures for fuzzy sets. Inf. Sci. **67**(3), 209–228 (1993). <https://doi.org/10.1016/0020-0255(93)90073-U>
3. T. Kiss, J. Strunk, Unsupervised multilingual sentence boundary detection. Comput. Linguist. **32**(4), 485–525 (2006). <https://doi.org/10.1162/coli.2006.32.4.485>
4. V.D. Blondel, J.-L. Guillaume, R. Lambiotte, E. Lefebvre, Fast unfolding of communities in large networks. J. Stat. Mech. Theory Exp. **2008**(10), P10008 (2008). <https://doi.org/10.1088/1742-5468/2008/10/P10008>
5. M. Dunaiski, W. Visser, J. Geldenhuys, Evaluating paper and author ranking algorithms using impact and contribution awards. J. Informetr. **10**(2), 392–407 (2016). <https://doi.org/10.1016/j.joi.2016.01.010>
6. S. Nie, S.R. Emory, Probing single molecules and single nanoparticles by surface-enhanced Raman scattering. Science **275**(5303), 1102–1106 (1997). <https://doi.org/10.1126/science.275.5303.1102>
7. E.C. Le Ru, E. Blackie, M. Meyer, P.G. Etchegoin, Surface enhanced Raman scattering enhancement factors:   a comprehensive study. J. Phys. Chem. C **111**(37), 13794–13803 (2007). <https://doi.org/10.1021/jp0687908>
8. M.F. Mrozek, M.J. Weaver, Detection and identification of aqueous saccharides by using surface-enhanced Raman spectroscopy. Anal. Chem. **74**(16), 4069–4075 (2002). <https://doi.org/10.1021/ac020115g>
9. K.E. Shafer-Peltier, C.L. Haynes, M.R. Glucksberg, R.P. Van Duyne, Toward a glucose biosensor based on surface-enhanced Raman scattering. J. Am. Chem. Soc. **125**(2), 588–593 (2003). <https://doi.org/10.1021/ja028255v>
10. H.Y.H. Chan, C.G. Takoudis, M.J. Weaver, Oxide film formation and oxygen adsorption on copper in aqueous media as probed by surface-enhanced Raman spectroscopy. J. Phys. Chem. B **103**(2), 357–365 (1999). <https://doi.org/10.1021/jp983787c>
11. S. Zou, M.J. Weaver, Surface-enhanced Raman scattering of ultrathin cadmium chalcogenide films on gold formed by electrochemical atomic-layer epitaxy:   thickness-dependent phonon characteristics. J. Phys. Chem. B **103**(13), 2323–2326 (1999). <https://doi.org/10.1021/jp990107c>
